# A generalised framework for detailed classification of swimming paths inside the Morris Water Maze

**DOI:** 10.1038/s41598-018-33456-1

**Published:** 2018-10-10

**Authors:** Avgoustinos Vouros, Tiago V. Gehring, Kinga Szydlowska, Artur Janusz, Zehai Tu, Mike Croucher, Katarzyna Lukasiuk, Witold Konopka, Carmen Sandi, Eleni Vasilaki

**Affiliations:** 1 Department of Computer Science, University of Sheffield, Sheffield, UK; 2 Laboratory of Epileptogenesis, Nencki Institute of Experimental Biology, Warsaw, Poland; 3 Neurobiology Center, Nencki Institute of Experimental Biology, Warsaw, Poland; 4 School of Computing, University of Leeds, Leeds, UK; 5 Laboratory of Behavioral Genetics, Brain Mind Institute, EPFL, Lausanne, Switzerland

## Abstract

The Morris Water Maze is commonly used in behavioural neuroscience for the study of spatial learning with rodents. Over the years, various methods of analysing rodent data collected during this task have been proposed. These methods span from classical performance measurements to more sophisticated categorisation techniques which classify the animal swimming path into behavioural classes known as exploration strategies. Classification techniques provide additional insight into the different types of animal behaviours but still only a limited number of studies utilise them. This is primarily because they depend highly on machine learning knowledge. We have previously demonstrated that the animals implement various strategies and that classifying entire trajectories can lead to the loss of important information. In this work, we have developed a generalised and robust classification methodology to boost classification performance and nullify the need for manual tuning. We have also made available an open-source software based on this methodology.

## Introduction

The Morris Water Maze (MWM), designed by Richard Morris, was first described in 1981 in a study regarding the spatial localisation of rats^1^. The MWM quickly became popular and by the end of the eighties a large number of published work using the MWM had been reported^2^. For instance, the review work of D’Hooge and Deyn mentions more than 2000 publications related to the MWM task within the decade 1990–2001^3^. More recently, virtual forms of the MWM have been used directly on human subjects and this generalisation made it possible to comparatively assess human and rodent place navigation^4^, compare spatial learning between sexes^5^ and directly study how certain factors (e.g. stimuli, age, etc.) affect spatial navigation and how certain areas of the brain perform under their effects^6–9^.

In a typical MWM experiment the rodent is placed inside a circular pool filled with water and is tasked with finding a hidden platform, which is placed in one of the four quadrants of the pool. Since the animal is unable to see the platform, it has to rely on external visual cues in order to navigate within the pool and find the platform. After a number of trials, it is expected that the animal will have learned the location of the platform and will therefore be able to find it in less time than in the beginning of the trials^10^.

Most of the studies using the MWM experiment utilise several measurements of performance in order to assess learning and memory. Many of these measurements have also been used to ensure that the animal groups have equal skills and abilities (e.g. swimming ability, speed, ‘understanding’ of the escape mechanism)^11,12^. Common measurements include the time that the animal spends inside each quadrant of the pool, the latency of finding the platform in each trial, the directionality and the total swimming distance in each trial^2,10,13^. There are also a number of more sophisticated measurements such as the body temperature of the animals throughout the experiment^14^ or the cumulative distance to platform, which is the distance between the animal location and the platform location calculated a number of times with a specific sampling rate^15,16^.

These simplistic measurements and statistics have been criticised as being insufficient to capture all of the different animal behaviours that are observed during MWM experiments^16,17^. For this reason researchers started to study the various behaviours that the animals were expressing inside the pool, which are known as exploration strategies. Notable are the studies of Wolfer *et al*., who computed a large amount of measures for each swimming path inside the maze in order to categorise the various strategies^18–20^. Other studies include the automatic classification procedures of Graziano *et al*.^21^ and Garthe *et al*.^22^, both of which specified regions of interest inside the arena. The categorisation method of Graziano *et al*. was based on a number of path measures while in the work of Garthe *et al*. a hierarchical classification algorithm was used and the categorisation of each swimming path was primarily based on the amount of time that the animal spent in each region of the arena. The latter method was also used in more recent studies^23,24^ (Illouz *et al*.^25^ proposed a classification technique based on support vector machines (SVM)^26^. While they trained their algorithm to detect nine behavioural strategies, their method was generic enough to detect these behavioural strategies on a variety of different MWM experiments. However, similar to previously proposed classification methods, it did not have the ability to detect mixed animal behaviours within the same trial but assigns the whole swimming path of the animal during the trial into one behavioural class^17^.

In our previous work^17^ we argued that animals employ several behavioural strategies during each trial in order to find the platform and assigning whole animal trajectories to single behavioural classes results in the loss of important information. For this reason, we proposed a more sophisticated automatic quantification methodology capable of classifying and presenting the various animal behaviours in much more detail during each trial. According to this approach, the animal swimming path is first split into segments and then the segments are classified into behavioural strategies. In this way, changes in the animal behaviour within each trial can be detected and the animal swimming path, as a whole, falls under more than one strategy, revealing how the animal behaviour evolves within the trial.

For the classification of the segments we used a semi-supervised classification procedure which requires manual classification (labelling) of a small amount of data. An advantage of this procedure is that our classification is based on a clustering algorithm which is able to detect patterns in the data. Therefore, the behavioural classes didn’t necessarily have to be defined a priori. On the other hand, the method developed in our previous study required a certain degree of knowledge about machine learning methods, which prevented the direct application to other datasets.

In this work, we present an automatic boosted classification procedure based on majority voting, which improves on the classification error, and a validation framework which leads to conclusions with a high degree of confidence. Majority voting refers to the fact that more than one classifier are used in order to assign a segment into a class. We have implemented this framework into a fully working software capable of performing all of our analyses, without requiring machine learning knowledge from the user. This software is called RODA (ROdent Data Analytics)^27^ and is focused on the MWM experiment. It provides an easy to use graphical user interface (GUI) for loading the data and defining the experimental specifications. It also supports automatic segmentation and semi-automatic classification, and produces quality figures which can be exported into various image formats.

## Results

### Trajectory Segmentation Analysis (TSA) & the RODA Software

We have developed a framework that allows Morris Water Maze trajectory segmentation analysis that requires little input from the user. Trajectories are divided into overlapping segments, a percentage of which (8–12%) are labelled by an expert user as belonging to one of eight different behavioural strategies. Multiple labels can also be used for a segment (see Methods for more information about the behavioural classes). The remaining segments are automatically classified via a semi-supervised clustering algorithm to one of the user-defined strategies, and via a smoothing procedure are mapped back to the full trajectories. This procedure allows us to identify multiple strategies in a single trial.

The user, in addition to providing labels, needs to define the segmentation length and overlap. For the segmentation parameters, appropriate regimes have been identified for the MWM with dimensions from 2 up to 2.5 times the arena radius (see Results: Robustness across different segmentation configurations).

In order to reduce the required tuning from the user (an issue of our previous work of Gehring *et al*.^17^) and improve the objectivity of the classification, we employ ensembles of classifiers that vote to assign the segment to a strategy according to a simple majority voting rule.

**Figure 1:**
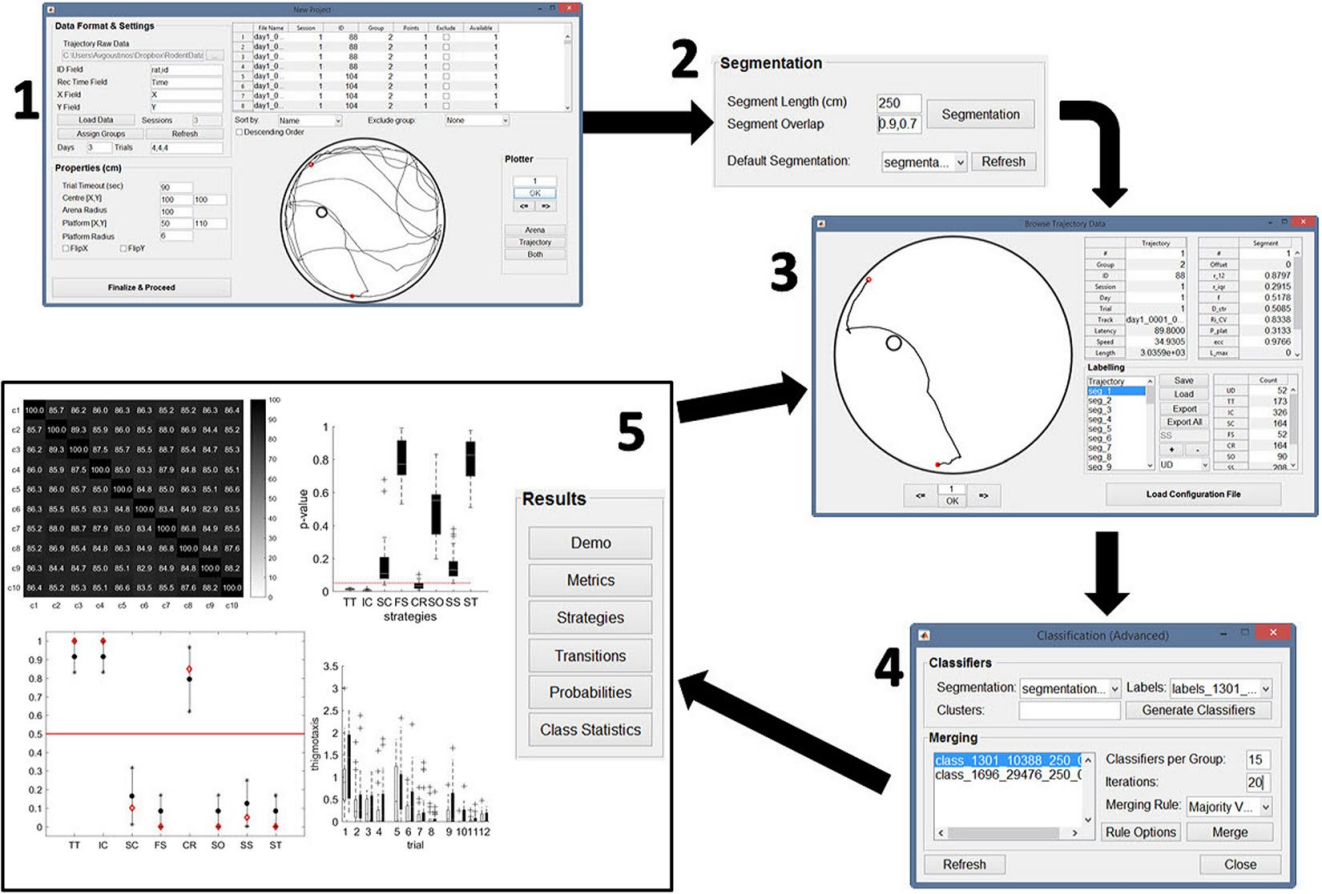
Screenshots of the software RODA. Each window is numbered to denote a separate stage of the workflow, which consists of: (**1**) the data input GUI, which is used to load the trajectory data extracted from Ethovision and select the specific tracks that will be used in the analysis; (**2**) the segmentation panel, which offers full control over the segmentation options; (**3**) the labelling GUI, which offers visualisation of entire trajectories and their segments allowing easy labelling of the segments; (**4**) the classification GUI, which contains options to tune various parts of the classification process (a default option is also available); (**5**) the results panel; which generates the analysis results. The results are generated in both graphical and textual formats. The user also has control over the output format of the image files as well as the elements of the generated figures such as text size, line width, etc. The arrow connecting (**5**) with (**3**) indicates that if the analysis results are not consistent then we need to go back to the labelling stage and provide additional or improved labels.

A software, called RODA^27^ (shown in Fig. 1), has been developed in order for our proposed framework to be available for usage by the scientific community. The software is available on the github repository https://github.com/RodentDataAnalytics/mwm-ml-gen under the GNU General Public License version 3 (GPL-3.0). A manual of the corresponding software can be found under the wiki section of the repository (https://github.com/RodentDataAnalytics/mwm-ml-gen/wiki) details on the methodology and technical information about RODA can be found under Materials and Methods.

### Advantages of Trajectory Segmentation Analysis (TSA)

Our methodology finds quantitative behavioural differences beyond those identified by standard metrics on the full swimming paths of the animals. It is able to detect additional significant differences between the behavioural strategies employed by the two or more animal groups in comparison to the categorisation of the whole animals trajectories. In more detail, from a manual behavioural analysis of the whole swimming paths of the animals the strategies thigmotaxis, incursion, scanning, self oriented, target scanning and direct finding are detected and analysed. By using TSA the additional behavioural classes of focused search, chaining response and scanning surroundings were able to be identified and analysed (for more details on the aforementioned classes of behaviour refer to Methods and Fig. 7).

**Figure 2:**
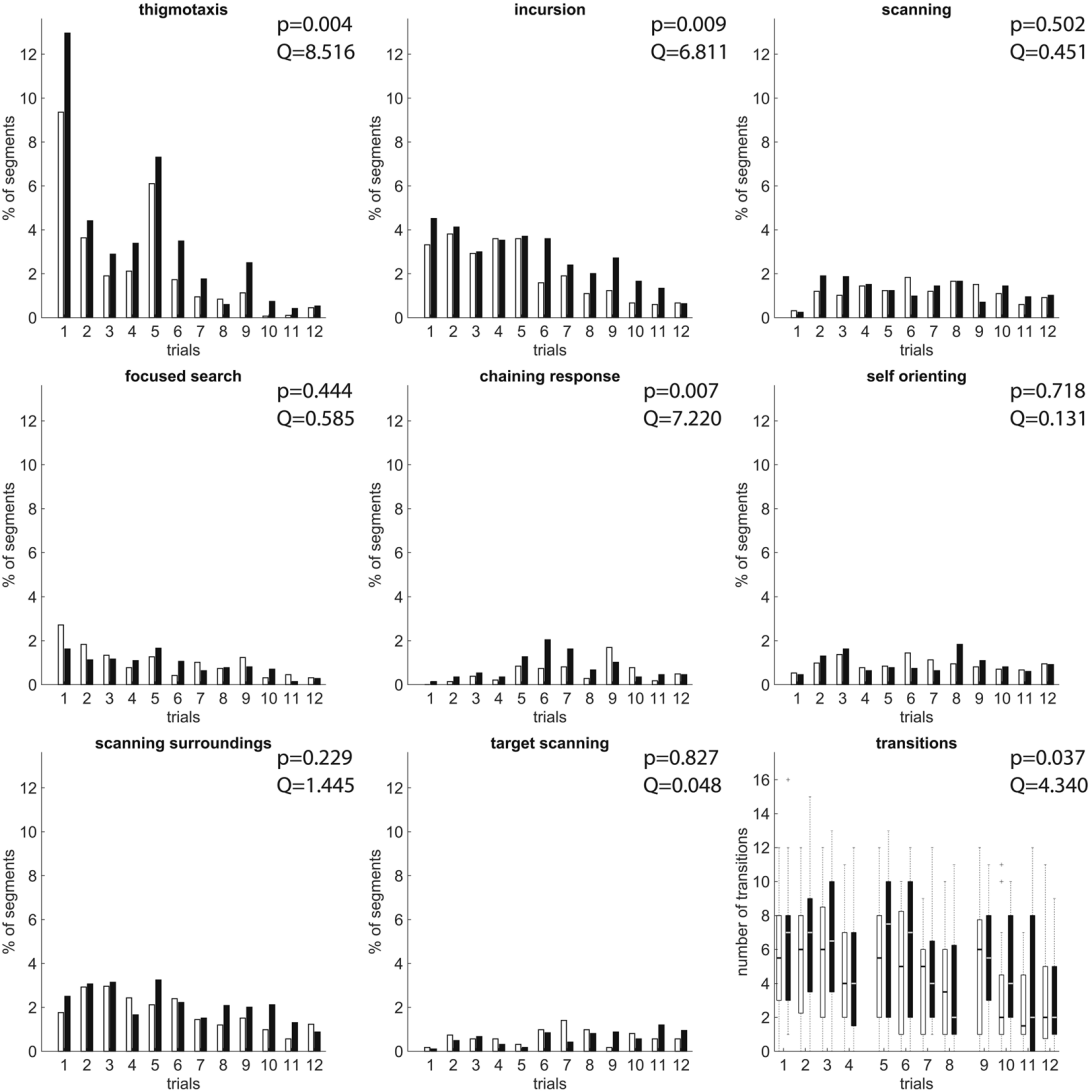
Percentage of segments falling under each strategy for the stressed (black) and control (white) animal groups over each trial. All the animals were tested for a set of 12 trials divided in to 3 sessions (days). Each segment (path interval; see Methods) is considered to be of length equal to the length of the arena radius (100 cm). For the transitions: bars represent the first and third quartiles of the data; the black (control group) or white (stressed group) horizontal lines are the medians, crosses are the outliers and whiskers indicate the minimum and the maximum values. These results were generated by using a segmentation length of 2.5 times the arena radius (250 cm) and 90% overlap; for the classification an ensemble of classifiers was created by using classifiers with validation error less than 25%. The Friedman test p-value (shown on the top right) was used to compare both animal groups for the complete set of trials. According to the plots Thigmotaxis and Incursion strategies show a clear difference in favour of the stressed groups (Friedman test p-value = 0.004, *Q* = 8.516, *k* = 2 and p-value = 0.009, *Q* = 6.811, *k* = 2) along with Chaining Response (Friedman test p-value = 0.007, *Q* = 7.220, *k* = 2). The number of transitions between strategies shows that the stressed animals change their behaviour more often than control animals within single trials (Friedman test p-value = 0.037, *Q* = 4.340, *k* = 2). Segmentation analysis is able to distinguish more behavioural differences between the two groups in comparison with the classification of the full swimming paths (see Fig. 4), which are then consistent with the performance measurements (see Fig. 3); stressed animals, despite running faster and sweeping longer swimming paths, require the same amount of time to detect the arena because they implement a series of inefficient strategies (i.e. Thigmatoxis and Incursion) or less effective strategies (i.e. Chaining Response). Furthermore they are switching behaviours (transitions) more often than the control animals indicating a loss of focus of finding the platform. The Direct Finding class was excluded from this figure because for this class the statistical analysis gives quantitatively the same results as in Fig. 4).

We applied our framework to the dataset of Gehring *et al*.^17^ composed of two rodent groups (stressed and control rats). The reason for selecting the same dataset was because we wanted a benchmark for our improved method and the ability to demonstrate its robustness and generality. The two animal groups differ on the strategies of Thigmotaxis (Friedman test p-value = 0.004, *Q* = 8.516, *k* = 2), Incursion (Friedman test p-value = 0.009, *Q* = 6.811, *k* = 2) and Chaining Response (Friedman test p-value = 0.007, *Q* = 7.220, *k* = 2) in favour of the stressed group meaning that stressed animals implement these strategies more often than the control group. In addition, stressed animals tend to transit between different strategies more often than the control animals (Friedman test p-value = 0.037, *Q* = 4.340, *k* = 2). For relevant results refer to Fig. 2.

**Figure 3:**
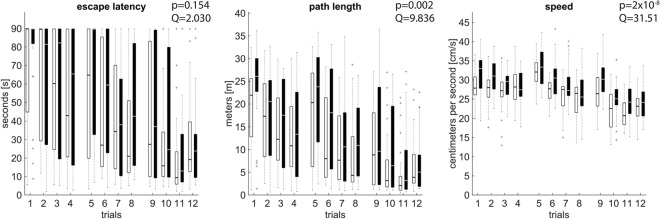
Full swimming path standard metrics for the stressed (black) and control (white) animal groups. All the animals were tested for a set of 12 trials divided in 3 sessions (days). Bars represent the first and third quartiles of the data; the grey line that splits the bars represents the median, crosses are the outliers and whiskers indicate the minimum and the maximum values. The Friedman test p-value over the trials is shown on the top right of each plot. Stressed animals find the platform as fast as the control group (escape latency, p-value = 0.154, *Q* = 2.030, *k* = 2) even though they run faster (path length, p-value = 2 × 10^−8^, *Q* = 31.510, *k* = 2) and sweep (on average) longer swimming paths (speed, p-value = 0.002, *Q* = 9.836, *k* = 2) within the trials than the control group.

**Figure 4:**
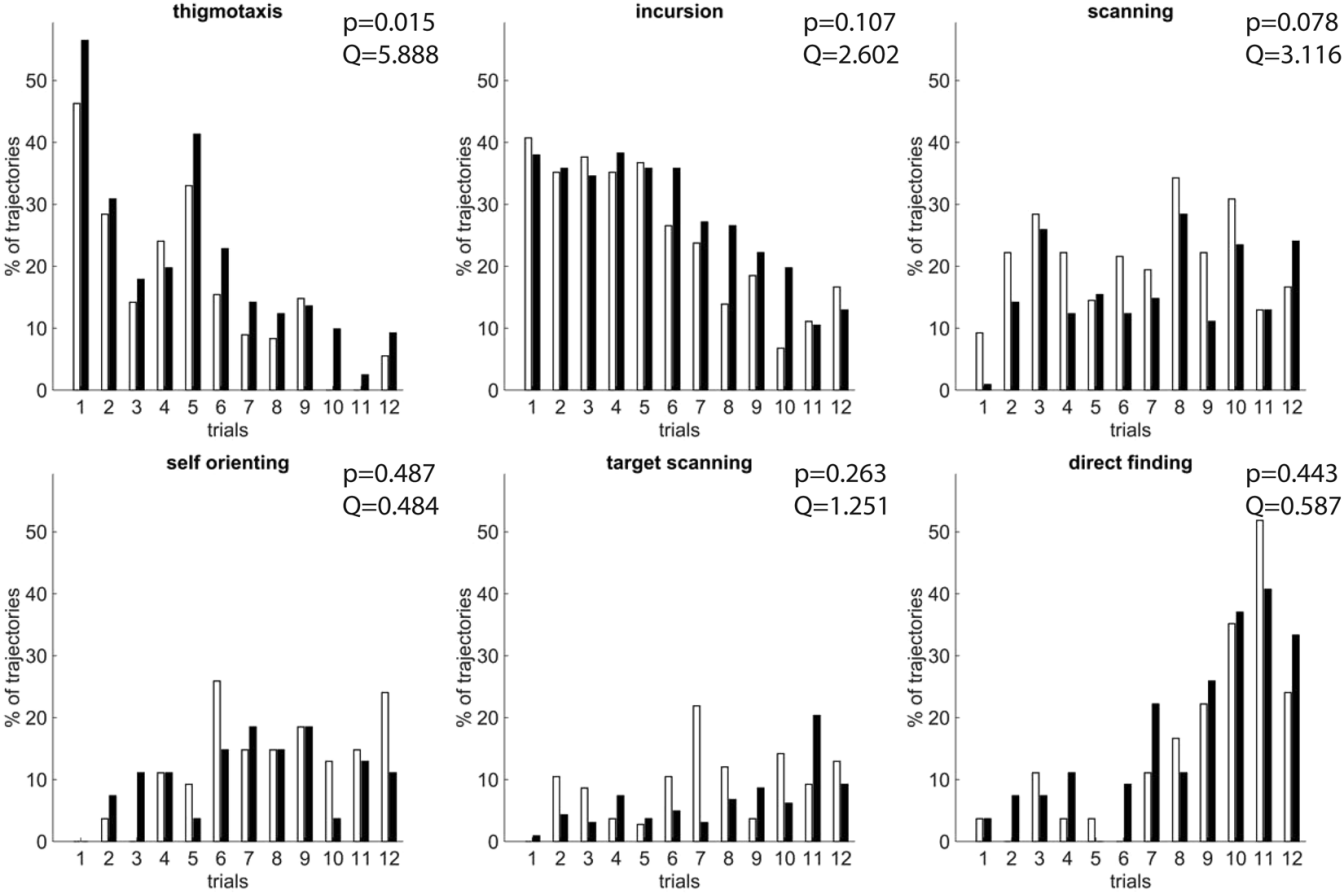
Manual classification of the full swimming paths. White bars: control group; Black bars: stressed group; the two groups were compared over the complete set of trials using the Friedman test (shown on the top right corner of each graph). In the manual classification of the full swimming paths, certain behavioural classes (Focused Search, Chaining Response and Scanning Surroundings) couldn’t be identified. Significant difference (Friedman test p-value = 0.015, *Q* = 5.888, *k* = 2) was detected only for the Thigmotaxis strategy in favour of the stressed animal group, indicating that stressed animals are implementing it more often than the control animals and have less chances of detecting the platform. This is relevant to the performance measurements (see Fig. 3) where stressed animals run faster and sweep longer swimming paths, but still fail to find the platform in less time than the control group. For more information about each behavioural strategy refer to Methods.

Commonly used measurements of learning (animal speed, escape latency and path length) suggest that there is a significant difference among the two animal groups in the sense that the stressed animals are faster (Friedman test p-value = 2 × 10^−8^, *Q* = 31.510, *k* = 2) and swap longer paths (Friedman test p-value = 0.002, *Q* = 9.836, *k* = 2) within the trials but they still fail to find the platform in less time than the control animals (Friedman test p-value = 0.154, *Q* = 2.030, *k* = 2). For relevant results refer to Fig. 3 for the relevant results). Manual classification of the full swimming paths to different behavioural strategies was performed due to the small amount of data; this analysis suggests that the reason for this phenomenon is because stressed animals tend to use the low level strategy of Thigmotaxis more than the control group (Friedman test p-value = 0.015, *Q* = 5.888, *k* = 2), which lowers their chances of finding the platform since they spent most of the time close to the arena periphery (refer to Fig. 4 for the relevant results). TSA agrees on that conclusion but it is also able to detect that stressed animals tend to use *a series* of low level strategies, both Thigmotaxis (Friedman test p-value = 0.004, *Q* = 8.516, *k* = 2) and Incursion (Friedman test p-value = 0.009, *Q* = 6.811, *k* = 2, refer to Fig. 2), which lower their chances of finding the platform since they spent most of the time on *or close to* the arena periphery. In addition, stressed animals implement the Chaining Response strategy more often than the control animals (Friedman test p-value = 0.007, *Q* = 7.220, *k* = 2, refer to Fig. 2), which implies that they haven’t memorised the location of the platform but its distance to the wall^18^; so they swim at that distance in hope to find it by chance; a behaviour that is again, on average, time consuming. Furthermore, TSA allows us to detect that stressed animals change their behaviour inside the arena more often than the control animals (Friedman test p-value = 0.037, *Q* = 4.340, *k* = 2, refer to Fig. 2). These results are relevant to studies such as^28–30^ which suggest that high levels of stress lead to weak attention and frequent behavioural switches.

### Robustness across different segmentation configurations

It is expected that the segmentation length affects the results, i.e. a full trajectory will not reveal more than one strategy or a very small segment will not have enough information for mapping it onto a strategy. We therefore focus on segmentation lengths between 2 times and 3 times the arena radius in order to investigate the robustness of our process.

**Figure 5:**
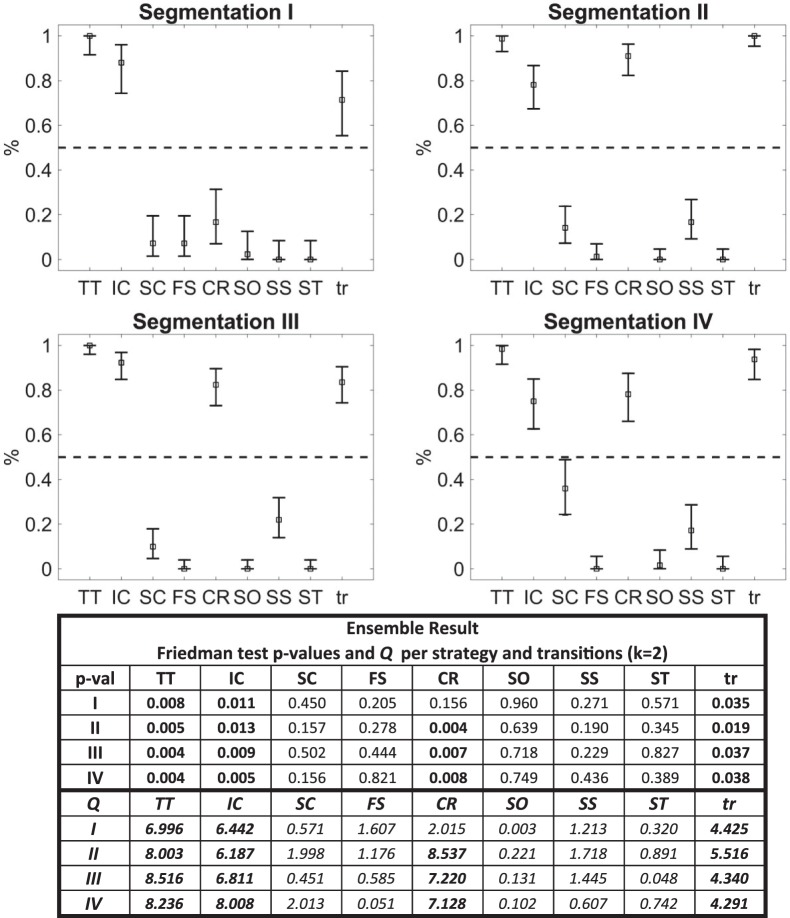
Conclusive results from the classification of each segmentation (see Table 1). Each plot shows the 95% binomial confidence intervals for the classifiers of each segmentation regarding their agreement on the significant difference between the two animal groups for each strategy and the strategy transitions. Squares indicate the mean of the classifiers; errorbars represent the 95% confidence intervals; the dashed line indicates the threshold of interest (0.5 or 50%). Confidence intervals clearly above 0.5 (or 50%) confirm that there is indeed a significant difference between the the two animal groups on the strategies and the strategy transitions. The table below the plots shows the Friedman test p-values (upper table) and the equivalent Friedman’s chi-square statistic (lower table) for the classification result of the ensemble; in all cases *k* = 2, control and stress columns. Segmentation configurations are arranged in columns and strategies in rows; each element has the relevant p-value and chi-square statistic and bold cells indicate significant difference, i.e. p-value < 0.05. Abbreviations: Thigmotaxis (TT), Incursion (IC), Scanning (SC), Focused Search (FS), Chaining Response (CR), Self Orienting (SO), Scanning Surroundings (SS), Target Scanning (ST), Strategy Transitions (tr) (refer to Methods for more information on each behavioural strategy). We see that in three cases (Segmentations II, III, IV) the two animal groups show significant differences in the strategies of Thigmotaxis (TT), Incursion (IC) and Chaining Response (CR) and transition between strategies (tr). We can see that while Segmentations II, III, IV agree that there is significant differences on the Thigmotaxis, Incursion, Chaining Response and transitions, Segmentation I fails to capture the significant difference on the Chaining Response because of the lengthy segments which caused this strategy to be overshadowed by other strategies and disappear (refer also to Table 3).

The animal swimming paths inside the maze were segmented using four different segmentation configurations (different segment length and/or segment overlap). For each segmentation, we provided labels to approximately 10% of the segments (refer to Table 1 for a summary of our different configurations) and we use our framework to classify the rest. We based our conclusions on both the ensemble classification result as well as the percentage of classifiers in an ensemble that agree to this result (95% binomial confidence intervals clearly above 50%). Three out of four segmentation configurations (with segment lengths 2 and 2.5 times the arena radius) led to the conclusion that the two animal groups (stressed and control) have significant difference on the strategies of Thigmotaxis, Incursion and Chaining Response and strategy transitions (Friedman test p-value < 0.05 and 95% binomial confidence intervals clearly above 50%, see Fig. 5 for detailed statistics) in favour of the stressed group meaning that stressed animals implement these strategies and transit between different strategies more often than the control animals. One out of four segmentations (segment length of 3 times the arena radius) failed to capture significant difference in the Chaining Response strategy and a probable reason is that the segment length is too large, thus strategies that are rarer and significantly smaller are overshadowed by more common ones (e.g., Chaining Response may be overshadowed by Scanning Surrounding or Thigmotaxis, refer to Table 3). This is an issue introduced already during the labelling procedure. For example, the Segmentation 1 only 0.67% of the samples were single-labelled as *chaining response* vs 1.58%, 0.72%, 1.06% for the Segmentations 2 to 4 correspondingly. The larger segment makes it more difficult for the human expert to distinguish rare classes that are adjoint to frequent ones.

**Table 1:** Parameters for the classification of four different segmentation configurations with variable segment lengths and overlaps.

	Segmentation I	Segmentation II	Segmentation III	Segmentation IV
Segment Length (cm)	300 (3 · *R*)	250 (2.5 · *R*)	250 (2.5 · *R*)	200 ($2\cdot R$)
Segment Overlap	70%	70%	90%	70%
Number of Segments	8847	10388	29476	13283
Number of Segments Labelled	988 (12%)	1261 (12%)	2445 (8%)	1227 (9%)
Total number of labels	1022	1313	2568	1232

For each segmentation a percentage of segments (between 8% and 12%) was manually labeled. Multiple labels could be given to each segment; in this study no more than two labels were given simultaneously to a segment. The segment length was selected to be proportional to the arena radius (*R*), which was equal to 100 cm. The segment overlap was used to avoid any unfavourable segmentation (see Methods).

## Discussion

Methodologies that classify swimming paths in MWM to behavioural classes can reveal different stages of learning in animal groups. However, up to now, there are very few examples of earlier research that have made use of machine learning techniques to automatically detect animal behaviours. Most of them have proposed methods that are difficult to generalise and require machine learning knowledge. In our previous study^17^ we addressed limitations of the previous techniques by focusing on the fact that forcing whole swimming paths into a single class of behaviour can be suboptimal as each trajectory incorporates a number of different behaviours. Our methodology of detailed trajectory classification can reveal additional behavioural differences between two groups of animals and can be used even when small amount of trajectory data are available since the segmentation process, due to overlapping, typically creates a significant amount of data. Nevertheless, our previously proposed method of segmented trajectories classification required a certain degree of machine learning knowledge to be used correctly, and allowed an amount of subjectivity when choosing classifiers.

In this work, we address these issues by proposing to improve the robustness of the technique via majority voting. Our results are no longer based on a single classification tuning (classifier) but on the agreement of many. This technique alleviated the subjective assignment of the swimming path segments to classes since, in practice, many classifiers that seemingly perform equally well in validation, have relatively high disagreement, and how to best chose among them might be unclear. Here, we systematically investigate different segmentations to identify what are the bounds under which our method produces meaningful results. The bounds refer to the minimum and maximum segmentation length and the number of labels that needs to be provided. Furthermore, the binomial confidence intervals on the ensemble of the classifiers are informative regarding the quality of our results.

The dataset from our previous work^17^ was used as a benchmark of our new methodology and also as a way to demonstrate its robustness and generality. We report that it leads to results similar to our earlier work^17^ but with one difference; we do not detect any significant difference for the scanning strategy which we had detected before based on the result of a single classifier. This is due to a number of factors: (i) the use of only one classifier, which results in higher error (see also the confidence intervals in Fig. 5), (ii) the merging of three different segmentations that resulted in classifications that didn’t fully agree with each other. Here we base our conclusions on the majority voting of many classifiers that are shown to have an improved performance versus the simple classifiers, and therefore lead to more reliable results.

One important point that should be mentioned is that despite the fact that for each segmentation the ensemble formed has extremely low to zero error percentage, the largest segmentation failed to indicate difference on the Chaining Response strategy. We have identified as cause of this issue the difficulty involved when labelling large segments; in this case the chaining response can be masked by more dominant classes such as Thigmotaxis. It is worth noting that the smoothing function, which is used to map the segments back to the whole trajectories, again do not affect the conclusions formed based on the strategies. Even without the smoothing function, again, three segmentations agree on the differences between the two animal groups on the Thigmotaxis, Incursion and Chaining Response strategies while the segmentation with the more lengthy segments cannot capture the difference on Chaining Response (refer to the Supplementary material for the non-smoothed classification results). For this reason, the criterion for correct classification cannot be based on the classification error alone. We also require consistent results within a reasonable variation of the segmentation length, in our case 200–250 cm, i.e., 2 R and 2.5 R, with R being the radius of the maze. We have also verify that these segmentation parameter arranges are directly applicable to other MWM experiments (results not shown).

To facilitate the use of this methodology by the scientific community, we provide a complete software incorporating of our framework which includes a Graphical User Interface (GUI) to guide the user throughout all the analysis stages and allows for the manual configuration of each procedure.

Though our proposed framework is able to detect behavioural information in much detail we should highlight that it has a substantial limitation; the segmentation length will inevitably have an effect on the behavioural strategy length. For instance, it is not able to detect behavioural strategies of lengths shorter than $2\cdot R$ (refer to the supplementary material, where we provide the average length of each behavioural class for all the segmentation tunings). Thus if groups have different strategy lengths, e.g. if only one group is having lengths more than $2\cdot R$ then our current method is still applicable but it will not provide much information about behavioural differences because most of the segments will be classified as Direct Finding. Main reasons for this limitation are the following: (a) it is difficult to put manual labels to segments with lengths below $2\cdot R$ and (b) trajectories with lengths below the length of the segmentation tuning will be automatically classified as Direct Finding. To alleviate this limitation we gave the user the ability to provide labels to trajectories shorter than the specified segmentation tuning. However, for other experimental data if the strategies are of average length below $2\cdot R$, the tuning procedure we presented here must be repeated to identify appropriate values.

A proposed future application of our current framework is to address differences in sequence of behavioural strategies. As it was suggested in the literature^31,32^, strategies within one trial occur in reliable sequences. Since the output of our method are sequences of strategies, one could potentially apply a Markovian analysis^33^ on the sequences and detect differences between animal groups on the probabilities of transition between behavioural strategies. A more detailed analysis on the different kinds of transition has the potential to reveal additional differences among animal groups. Such analysis can be viewed as a Markov model where each behavioural strategy is a state and when the animal is in a particular state it can either remain in the same state, i.e. repeat the same behaviour, or transit to another behaviour.

Finally, it should be noted that the work we present here can generalise to other species of rodents inside the MWM (e.g. mice) as well as other experiments similar to the MWM (e.g. open field tasks, place avoidance). Two main significant changes to be made are the strategy definitions and the trajectory features. In our recent work^34^ we addressed the issue of pre-defined strategies by using a fully unsupervised procedure to find patterns of behaviour in the active allothetic place avoidance task. In that experiment there is no previous knowledge of animal behaviours thus supervised or semi-supervised techniques cannot be applied. However, we mentioned that our classification depends on the trajectory features that we used. A combined work of the classification boosting technique, an unsupervised methodology^34^, and the engineering of trajectory features that not linked to a specific experiment has the potential to lead to a robust generalised framework of trajectory analysis for many different animal species used in experimental procedures (e.g. octopus^35^ and zebrafish^36^).

## Methods

### Analysis Overview

In our proposed analysis method, the swimming paths of the animals inside the Morris Water Maze are divided into segments of approximately equal length and a fixed overlap percentage. For each segment a set of eight features is computed (refer to the Supplementary material for a short description of each feature). The features are then used in the classification procedure. Finally, a small portion of the segments needs also to be assigned manually to a specific strategy (labelling); this information is used as prior knowledge to guide the classification procedure.

Our classification procedure, which assigns segments to classes of behaviour, is based on a semi-supervised clustering algorithm called Metric Pairwise Constrained K-Means (MPCK-Means)^37^. This algorithm incorporates the two main approaches of semi-supervised clustering: metric learning (the measuring of similarity, ‘distance’, between data) and constrained-based learning (the use of labels or constraints that produce a better grouping of the data). To turn the algorithm into a classifier, the labelled data were used not only to guide the clustering process but also to assign clusters to classes (see Supplementary material).

A common issue with many clustering algorithms, including MPCK-Means, is that a predefined number of target clusters needs to be provided; this number indicates the number of clusters into which the data will be partitioned. Determining the optimal number of target clusters is challenging and, although many different quality measures were proposed over time^38^, this value will depend on the specific clustering method and data at hand.

**Figure 6:**
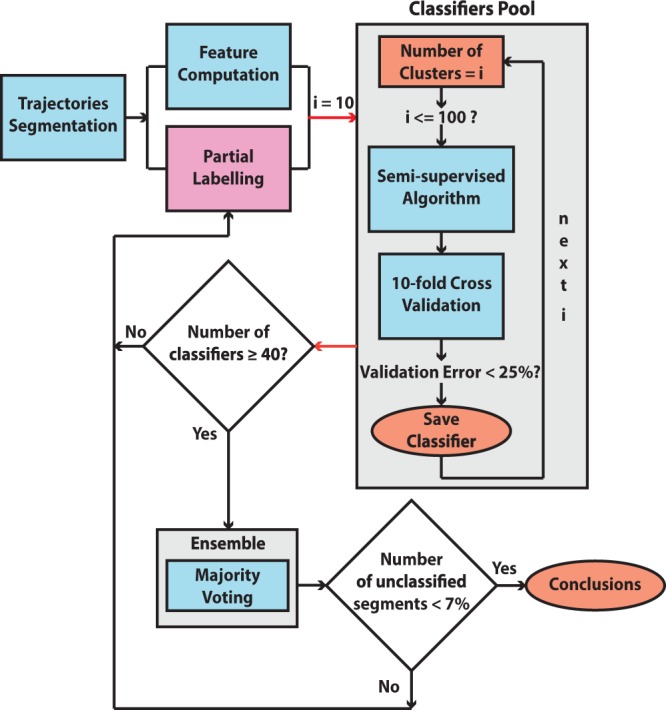
Workflow diagram illustrating the analysis procedure. Cyan boxes indicate automatic process; orange boxes indicate objects of importance or results; Partial Labelling box (magenta) implies extensive user interaction with the process; the grey box groups the processes taking part in the classification procedure. After the trajectory segmentation, eight trajectory features for each segment are computed and a certain number of segments are manually labelled. Afterwards a pool of classifiers is generated. ‘Strong’ classifiers (cross validation error <25%) are then selected from the pool and work together (majority voting) as a team (ensemble) to produce the classification results. Throughout the process the labelling quality is constantly assessed and in case of weak classification results we go back to the labelling stage.

In this work, instead of searching for an optimal number of clusters and attempting to generate an optimal classifier, we select to generate a pool of ‘strong’ classifiers whose ‘goodness’ is assessed based on the 10-fold cross validation error. The strong classifiers generated in that way are then used to form an ‘ensemble’ which uses majority voting to reach a classification decision. The two conditions of having both strong and diverse classifiers are essential in majority voting in order to reach an optimal classification solution^39,40^. This will be discussed in more detail later. In order to assess the labelling procedure (if enough and consistent labels have been provided) the criterion of having a minimum of 40 strong classifiers has been added prior to majority voting. Finally, the classification result of the ensemble is expected to have a low percentage of unclassified segments (less than 3%) because, since the classifiers are diverse, they will have different errors or will fail to classify different segments. Thus if they work together and form an ensemble, the individual errors will be compensated by the correct responses of the other members of the ensemble^39^. A diagram of the procedure is illustrated in Fig. 6.

### Trajectories Segmentation and Partial Labelling

To assign one trajectory to multiple classes, we earlier proposed the division of the full animal swimming paths into segments^17^. In our method each segment overlaps significantly with its previous one (percentages of 70% and 90% have been performed on this analysis) to make sure that important information is not lost due to an unfavourable segmentation. The segment length was empirically selected to be equal to, or slightly longer than, one arena diameter. If the segment length is too short it might be difficult to identify to which class segments belong; if it’s too long it might happen that more than one class of behaviour is represented. The latter case can be seen in our results, where the large segment length (3 times the arena radius) causes some classes to be overshadowed by the more common classes (refer to Fig. 5).

In this study, nine predefined strategies were adopted (see Classes of Behaviour). We have found empirically that the amount of data that needs to be labelled should be roughly between 8% to 12% of the total segment number but the exact value depends greatly on the dataset under investigation. As a rule of thumb, if fewer labels are provided then the classification results will be poor in the sense that a lot of segments will remain unclassified or fall under the wrong class. Since the labelling procedure is prone to error and subjectivity a number of validation criteria have been implemented throughout our analysis.

### Classification Boosting with Majority Voting

The classification boosting is an ensemble technique that is based on the idea that many weak learners can be converted to a strong learner^41^. In machine learning terms an ensemble of weak classifiers (classifiers that make mistakes) can be used to form a strong classifier (classifier that makes fewer mistakes) by combining each individual’s opinion^42,43^. This approach has been used in various classification tasks (see Oza *et al*.^44^ for a survey) and in addressing complex real-world problems, when single algorithmic classification solutions are unable to achieve high performance^45^.

One way to perform classification boosting is through majority voting^42^: many classifiers form an ensemble, vote for the class of each datapoint and the class with the most votes wins. The output of the ensemble is expected to have improved accuracy since individual errors of each classifier are compensated by the correct responses of the other members of the ensemble^39^. In order to achieve such an outcome, the classifiers need to at least be diverse in the sense that they should not share the same errors^42,46^. It should be noted, however, that diversity alone is insufficient to ensure that randomly selected, arbitrarily weak, classifiers will achieve high classification accuracy^39^ and^47^. Individual Classifiers have to also be strong meaning that they should be sufficiently accurate on their own^47^ (and^48^ indicate an accuracy of at least 50%).

#### Majority Voting Implementation

In our framework, we need to classify different trajectory segments into animal behavioural classes (strategies) having only a partial set of labelled data. The classification is parametrized by the target number of clusters of the clustering algorithm, a value that is difficult to estimate in advance. In order to overcome this problem we generate a number of classifiers by providing different numbers of target clusters in succession; At the end of this process a pool of classifiers is generated. We then use the 10-fold cross validation^49^ process to evaluate different numbers of target clusters (10 to 100). Only classifiers with a validation error lower than 25% are used to form an ensemble (for more information about the 10-fold cross validation procedure refer to the Supplementary material). We set the minimum number of required classifiers that fulfill this criteria to 40. The reasoning behind this process is that we require a sufficient number of ‘strong’ classifiers. For the majority voting, we adopt the simple scheme where the vote of each classifier has the same weight^50,51^ and that in case of a tie the datapoint (segment) is marked as undefined.

### Framework Validation

We thoroughly validated every procedure of our framework in terms of robustness and results consistency. Overall, we performed four different segmentations with the aim to find the bounds (error margins) for the segment length between which we have consistent analysis conclusions. As discussed in the results section, it is expected that the segmentation length affects the results and we showed that consistency for the MWM can be achieved with segmentation lengths between 2 times and 2.5 times the arena radius. For more information refer to Fig. 5 where we show that longer segment lengths fails to capture the difference between the two groups of the Chaining Response behavioural strategy.

For each of the four different segmentations we compared the performance of the classifiers, the ensemble and multiple ensembles formed by random sample of ‘strong’ classifiers. Table 2 shows the relevant results of the last stage of analysis, where the overlapped segments have been mapped back to the original swimming paths. For the latter the smoothing function is applied on the segments (refer to Methods: Mapping Segment Classes to the Full Swimming Paths) and this detail is important because the smoothing procedure increases the performance of the classifiers (for the statistical analysis prior to the smoothing function refer to the Supplementary material). As expected, ensembles have higher accuracy, a lower percentage of unclassified segments and a higher percentage of agreement among them in comparison to individual classifiers. However, since in our method the cross validation was used for both tuning and testing, additionally we manually assess the error of the ensembles on two out of the four segmentations (see Supplementary material for more information about the manual assessment).

**Table 2:** Classification statistics (average) for the four segmentation configurations of Table 1 and benefits of majority voting.

	Segmentation I	Segmentation II	Segmentation III	Segmentation IV
Number of generated Classifiers	42	78	91	64
	Performance: Classifiers			
Average Error (%) [min-max]	16.8 [5.4 24.9]	17.5 [3.7 25.0]	13.9 [1.8 21.5]	18.0 [7.3 24.9]
Unclassified (%) Segments	2.5	2.5	1.3	3.7
Agreement (%)	58.7	61.0	59.6	56.3
	Performance: Ensemble(s)			
Error (%)	0.0	0.2	0.0	0.0
Unclassified (%) Segments	0.0	0.0	0.0	0.1
Agreement (%)	83.4	82.6	82.3	80.0

(**1**) Number of generated classifiers: based on each segmentation, only classifiers with cross-validation error lower than 25% were selected to take part in the classification analysis procedures (ensemble and binomial confidence intervals). As a rule of the thumb we require a minimum number of 40 ‘strong’ classifiers to be generated in order to trust the classification results. (**2**) Error: the 10-fold cross validation was used in order to select ‘strong’ classifiers based on their validation error. 10-fold cross validation was also used to compute the average accuracy of the ‘strong’ classifiers and the accuracy of the ensemble (in case of the ensemble, the same folds used by the classifiers were re-used). The ensemble significantly benefits the classification accuracy. Since in our method the cross validation was used for both tuning and testing we manually assess the error of the ensembles on two out of the four segmentations (see Supplementary material). (**3**) The percentage of unclassified segments was computed separately; since the classifiers are ‘strong’ only a few segments remain unclassified, nevertheless the ensemble almost totally nullifies the unclassified segments. (**4**) The average agreement between the classifiers was computed by first calculating the percentage of agreement within each pair (we have agreement when two classifiers have assigned the same label on a particular segment) and then averaging all the agreements together (refer to Validity Measurements for more information). In order to perform the same statistical measurement in the ensemble domain, 21 ensembles were created by picking a random sample of 11 ‘strong’ classifiers from the pool. The agreement between the classifiers is better than moderate and, as expected, the agreement of the ensembles is high. We chose a sample smaller than 40 to avoid a large overlapping of classifiers across ensembles.

#### Classifier Diversity

To evaluate the diversity of the classifiers, we assess the percentage of their agreement for the class of each segment. The result is a symmetric matrix with rows and columns representing the classifiers where each element shows the percentage of segments for which two classifiers agree on the assigned class. The diagonal values of this matrix equal to 100 as each classifier is in 100% agreement with itself (refer to the Supplementary material for an example of an agreement matrix). An overall agreement can be computed by averaging the upper or lower triangular of the matrix. In addition, we consider the average cross validation error (accuracy) over the classifiers. In order for the classifiers to be both diverse and strong it is expected that they should have an average percentage of agreement well below 100% (in our case around 60%) and low cross validation error (refer to Table 2).

As has been previously reported^39^, ensembles have far less variance in comparison with individual classifiers thus it is expected to have much higher agreement. To demonstrate this observation, we generated a number of ensembles by picking classifiers at random from the pool. Afterwards we performed the same statistical measurement of agreement for the ensembles, similar to the one described for the classifiers. In contrast to the classifiers, the ensembles have high agreements among them (more than 80%) and nearly nullify the cross validation error of the classifiers (see Table 2). However, since in our method the cross validation was used for both tuning and testing^17^, additionally we manually assess the error of the ensembles on two out of the four segmentations (see the Supplementary material for the manual assessment results).

#### Percentage of unclassified segments

A useful measure for the quality of the classification is the percentage of unclassified segments. For certain segments, it is expected that none of the classifiers in the ensemble will be able to determine a class, or that there could be a draw for segments that transit between classes (refer to Table 3 on the Results section). This, however, does not have an impact on the consistency of results (see Fig. 5).

**Table 3:** Percentage of segments falling under each class for the four segmentation configurations of Table 1.

	Segmentation I	Segmentation II	Segmentation III	Segmentation IV
Thigmotaxis	27.7%	24.0%	24.6%	22.5%
Incursion	19.0%	18.9%	20.6%	17.0%
Scanning	10.2%	12.3%	10.5%	11.9%
Focused Search	9.2%	8.9%	8.2%	10.0%
Chaining Response	4.5%	5.8%	5.5%	9.8%
Self Orienting	7.1%	8.8%	8.2%	8.4%
Scanning Surroundings	17.4%	15.8%	16.8%	12.9%
Target Scanning	4.9%	5.6%	5.6%	7.4%
Unclassified	0.0%	0.0%	0.0%	0.1%

Some differences among the four segmentations are visible although based on the results of Fig. 5 consistency on the conclusions is preserved in segmentations II, III and IV. Regarding segmentation I, where there is no indication of any difference between the two animal groups regarding the Chaining Response strategy,;more segments are identified as Thigmotaxis and Scanning Surroundings. This indicates the possibility that some segments which transit between Chaining Response and one of these strategies are classified either as Thigmotaxis or Scanning Surroundings.

### Mapping Segment Classes to the Full Swimming Paths

The classification has been performed on overlapping segments of the animals’ swimming paths, we therefore need to map them back to the whole trajectories.

As a first approach, we considered the classified segments as continuous parts of the trajectories ignoring the overlap percentage. This method provides consistent results on the significant differences of the strategies but fails to detect differences on strategy transition between groups (refer to the Supplementary material for the relevant result). The reason for this is that sparse segments within each swimming path fall under different classes thus viewing them as a sequence leads to an overestimation of transitions (a transition occurs when a segment falls under a different class after a sequence of segments that fall under the same class).

To address this limitation, we use a smoothing technique with parameters independent of segmentation choice. This was done for two reasons: (i) to avoid subjective conclusions based on a specific segmentation configuration and (ii) to be able to directly compare different segmentations. In more detail, given that *R* equals to the radius of the arena, the swimming paths are now divided into intervals of length *R*. Each of the intervals is assigned to a certain class based on a weighed voting of all the overlapping segments. The mathematical expression for this operation is shown in equation 1,

1 $${C}_{{T}_{i}}\equiv ar{g}_{{c}_{k}}\,max\,\sum _{(\begin{array}{c}{S}_{j}\in {c}_{k}\\ {T}_{i}\cap {S}_{j}\ne \varnothing \end{array})}\,{w}_{k}\cdot {e}^{-\frac{{d}_{ij}^{2}}{2\cdot {\sigma }^{2}}}$$

where *T*_*i*_ is the *i*_*th*_ interval, *d*_*i*,*j*_ is the distance from the centre of the *j*_*th*_ segment (*S*_*j*_) overlapping with the *i*_*th*_ interval to the centre of the *i*_*th*_ interval, *c*_*k*_ is the *k*_*th*_ segment class and *w*_*k*_ is a class weight normalised so that $\sum \,{w}_{k}=1$. The sum is to be taken over the segments intersecting with the interval *T*_*i*_, belong to class *c*_*k*_ (unclassified segments are excluded) and fulfill the threshold requirement ${e}^{-\frac{{d}_{ij}^{2}}{2\cdot {\sigma }^{2}}} > \,=0.14$, where *σ* is the variance of the Gaussian and the value 0.14 is obtained when ${d}_{ij}=2\cdot \sigma$. The reason for the latest requirement is to create a cutoff for the segments that are too far away from the centre of the interval. The parameter *σ* controls the weight of the vote of each segment based on its distance from the interval and in our analysis it was set equal to *R* in order to achieve proportionality with the arena dimensions (other values have also been tested, refer to the Supplementary material). Finally, the class weight *w*_*k*_ was defined as ${w}_{k}=\frac{1}{P({c}_{k})}$, where *P*(*c*_*k*_) is the percentage of segments belonging to class *k*. The intuition for setting the class weights inversely proportional to the amount of segments that fall under each class was to prevent rare classes from being overshadowed by common ones. To prevent having too small or too large class weights the bounds of [0.01 0.5] were set, which means that if less than 1% or more than 50% of the segments fall under a certain category then this class will receive weight equal to 0.01 or 0.5 respectively.

### Statistics

The non-parametric Friedman test^52^ was used for the analysis of variance of each strategy between the two animal groups. This test was selected because the data are not normally distributed and because of its ability to control the variability among subjects over the different observations^53^.

For our analysis the null hypothesis is that there in no difference between the two animal groups (stressed and control) over each one of the strategies (refer to Methods: Classes of Behaviour) as well as over the number of times that the animals change their behaviour within single trials (strategy transitions). Small p-values (<0.05) generated by the Friedman test lead us to discard the null hypothesis that the results are identical and that any differences are only due to chance (random sampling). When the test is used we report the Friedman test p-value and the Friedman’s chi-square statistic (*Q*)^54^. Since we compare two animal groups, stress and control, we have *k* = 2 variables and the degrees of freedom are equal to *df* = 1.

In addition to the Friedman test, the 95% confidence intervals of a binomial distribution^55^ are being used, where the significance of a specific classification, as judged by each of the classifiers that form the ensemble, is viewed as a random process generating one (significant differences) or zero (non-significant differences). In more detail, the confidence intervals indicate our confidence that the classifiers forming the ensemble are on average pointing to the same conclusion as the ensemble (i.e. the majority agrees that there is significant difference over strategies or strategies transitions). Given that the Friedman test can have two outcomes, we hypothesise the outcomes to be the result of a binomial distribution. We require that the 95% confidence intervals to be clearly above 0.5 (or 50%) in order to be confident that the result in not due to chance^56,57^.

### The RODA Software

RODA^27^ consists of a series of graphical user interfaces (GUIs) which offer straightforward analysis of trajectory data extracted from the Noldus Ethovision System^58^. Every stage of the process can be tuned to meet the user’s needs. The generated figures can be exported into a variety of different image formats (JPEG, TIFF, etc.) while the numerical data depicted in the figures are also saved in Comma Separated Values (CSV) file format in case the user wishes to generate the figures using a different software (e.g. Microsoft Excel).

The software is entirely written in MATLAB^59^ and uses a modified version of the WEKA library^60^ written in Java which is known as WekaUT (for more information refer to http://www.cs.utexas.edu/users/ml/risc/code/).

The code of RODA is open-source and available on the github repository https://github.com/Rodent-DataAnalytics/mwm-ml-gen. The code requires the MATLAB’s Statistics Toolbox^61^ to be installed. Compiled versions of the software are also available for Windows and MAC OS (see the releases tab of the repository https://github.com/RodentDataAnalytics/mwm-ml-gen/releases).

### Classes of Behaviour and Strategy Transitions

**Figure 7:**
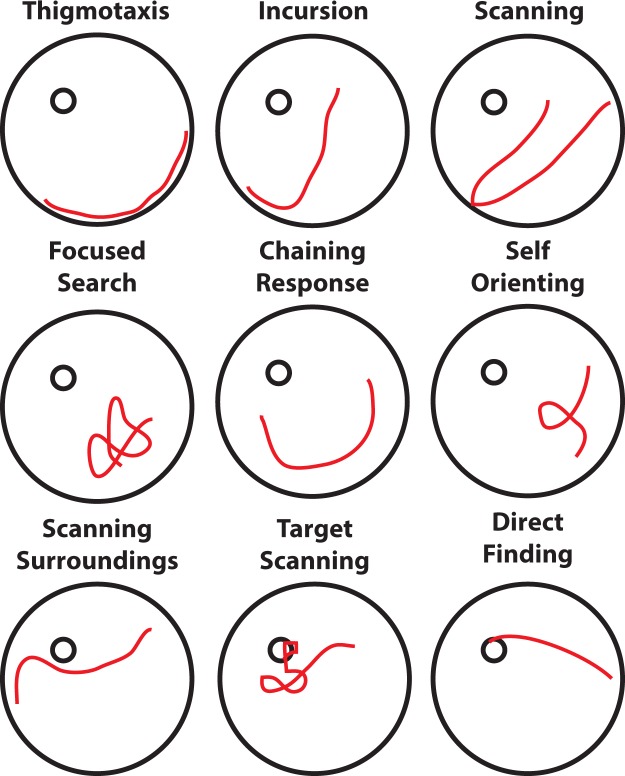
Stereotypical classes of behaviour. Each figure shows an example of a trajectory segment falling under each behavioural class. Throughout the experiment, the animals implement different strategies in order to solve the maze. By detailed analysis of each trial trajectory data into segments the interchange of these stereotypical animal behaviours becomes visible.

The choice of the classes of behaviours (strategies) in our analysis is motivated by previous studies (e.g.^19,21,62^) which have observed and reported stereotypical animal behaviours inside the MWM (for an example of each strategy refer to Fig. 7).

#### Thigmotaxis (TT)

The animal moves exclusively on the periphery of the arena and most of the time it touches the walls of the arena.

#### Incursion (IC)

The animal starts to distant itself from the arena periphery with visible inward movements.

#### Scanning (SC)

A behaviour associated with random searches focused in the centre of the pool. Another characteristic of this behaviour is that the animal rapidly turns away from the arena walls if it touches them^21^.

#### Focused Search (FS)

This behaviour is also associated with random searches but here the animal actively searches a particular small region of the arena.

#### Chaining Response (CR)

A behaviour first observed in the study of Wolfer *et al*.^18^ where the animal appears to have memorised the distance to the platform from the arena wall and swims circularly in order to find it.

#### Self Orienting (SO)

The animal performs a loop and orients itself inside the arena^21^.

#### Scanning Surroundings (SS)

The animal crosses a region very close to the platform of the arena but moves away^17^.

#### Scanning Target (ST)

The animal actively searches for the arena by swapping paths around it.

#### Direct Finding (DF)

The animal navigates straight to the platform.

#### Strategy Transitions (tr)

In addition to the behavioural strategies, we have analysed the number of times that the animals change their behaviour within single trials.

### Morris Water Maze Experiment and Data Properties

The data have been collected from experiments performed at the Laboratory of Behavioural Genetics, EPFL at Lausanne, Switzerland. All procedures were conducted in conformance with the Swiss National Institutional Guidelines on Animal Experimentation and approved by a license from the Swiss Cantonal Veterinary Office Committee for Animal Experimentation.

The water maze had a diameter of 200 *cm* with a submerged platform of diameter 12 *cm*. The recordings of the animals trajectories were performed by using the tracking software, Noldues EthoVision^58^ version 3.1. The dataset contains 57 rats, 30 of which were inducted into stress at peripubertal age^63^ and 27 of which were the control group. A total of 12 trials were performed per animal divided into 3 consecutive days with 4 trials per day. The timeout of each trial was 90 seconds and if the animal failed to find the platform within the time limit it was guided to it. The inter-trial interval between the trials of the same day was only a few minutes. The starting position of the animals was altered between trials.

## Electronic supplementary material

Supplementary material

## Data Availability

The data used in this work are available in the same GitHub repository that hosts RODA (https://github.com/Rodent-DataAnalytics/mwm-ml-gen). RODA has a demo function embedded for importing the data and reproducing the results of this work (see the Wiki section of the repository).
