# Supplementary material for: A generalised framework for detailed classification of swimming paths inside the Morris Water Maze
Source: Sci Rep. 2018 Oct 10;8:15089. doi: 10.1038/s41598-018-33456-1 (PMC6180070; doi:10.1038/s41598-018-33456-1)
Supplement: Supplementary file 1 — Supplementary material [file 41598_2018_33456_MOESM1_ESM.pdf]

Supplementary material for:
A generalised framework for detailed classification of swimming paths inside the Morris Water Maze

Avgoustinos Vouros[1,*], Tiago V. Gehring[1], Kinga Szydlowska[2], Artur Janusz[2], Zehai Tu[1], Mike Croucher[1], Katarzyna Lukasiuk[2], Witold Konopka[2], Carmen Sandi[3], and Eleni Vasilaki[1,+]

[1]Department of Computer Science, The University of Sheffield, Sheffield, UK
[2]Department of Molecular and Cellular Neurobiology, Nencki Institute of Experimental Biology, Warsaw, Poland
[3]Laboratory of Behavioral Genetics, Brain Mind Institute, EPFL, Lausanne, Switzerland
[*]avouros1@sheffield.ac.uk
[+]e.vasilaki@sheffield.ac.uk

A Trajectory Features

Table S 1. List of features used for the clustering procedure prior to the classification (see also our earlier work of[1]).

Feature name	Definition	Purpose
Eccentricity	$\varepsilon \equiv \sqrt{1-\frac{b^2}{a^2}}$, where a and b are the semi-major and semi-minor axes of the minimum enclosing ellipse.	Measure the elongation of the segments.
Focus	$f \equiv 1-\frac{4A}{\pi d^2}$, where A is the area of the minimum enclosing ellipse and d the segment length.	Measure how much the animal is searching a specific area of the arena.
Inner radius variation	$CV_{ri} \equiv IQR_{ri}/ri$, where ri is the median distance of every segment point to the center of the minimum enclosing ellipse and IQR_{ri} is the inter-quartile range of the inner radius.	Measures the relative dispersion of points related to a circle. The specific formula was used to increase the robustness and stability against outliers.
Maximum loop length	$MLL = \frac{d}{l}$, where d is the length of the longest self-intersecting loop and l is the total length of the segment. If no intersection is present, the value 0 is assigned.	It is used mainly as a measurement of the self-oriented movements
Central displacement	It is the distance d from the center of the minimum enclosing ellipse to the center of the arena divided by the arena radius.	It is used to identify concentric paths with the arena.
Median distance to center	The median of the distance of each datapoint to the center of the arena divided by the arena radius.	It is used to identify the amount of time that the animal spends next to the walls of the arena.
IQR distance to center	The IQR (inter-quartile range) of the distance of each datapoint to the center of the arena divided by the arena radius.	It is used to identify the spread of the time during which the animal is moving next to the walls of the arena.
Target proximity	Percentage of the path lying within an area centered at the center of the platform and radius 6 times the platform radius.	It is used to identify if the animal spends time actively searching for the platform or to capture random crosses through or close to the platform.

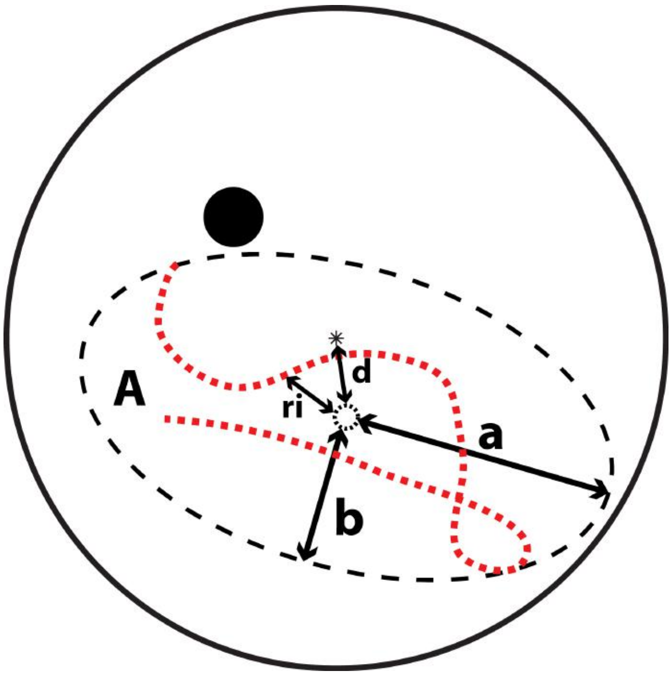

Figure S 1. Information about the minimum enclosing ellipse. The minimum enclosing ellipse is defined as the smallest enclosing ellipsoid of a set of points[2]. **Outer circle:** the Morris Water maze arena; **black circle:** the hidden platform; **star:** center of the arena; **red dots:** sample of points forming a trajectory segment; **dashed ellipse:** the minimum enclosing ellipse to the trajectory segment, **dashed circle:** the center of the minimum enclosing ellipse; **A:** area of the minimum enclosing ellipse; **a and b:** the semi-major and semi-minor axes of the minimum enclosing ellipse; **d:** distance between the center of the arena and the center of the minimum enclosing ellipse; **ri:** distance between the center of the minimum enclosing ellipse to the segment point i.

B Semi-supervised Classification Algorithm

Our classification algorithm is based on the Metric Pairwise Constrained K-Means (MPCKMeans) clustering algorithm implemented by Bilenko et al.[3].

MPCKMeans is inspired by the standard K-means[4] clustering algorithm and belongs to the family of semi-supervised algorithms. It is able to organise a set of datapoints into groups (clusters) according to their pattern similarities and it uses a set of labelled data (predefined knowledge) in the form of "CANNOT-LINK" and "MUST-LINK" constraints in order to improve the accuracy of assigning datapoints into clusters[1]. Moreover, it has the ability to create clusters of different shapes and sizes by using different metrics to minimize the distance between datapoints of the same cluster and to maximize the distance between datapoints of different clusters (features weighting)[3].

With the use of labelled data it is possible not only to guide the clustering procedure (with the creation of constraints) but to also combine clusters together and form larger groups (classes) which are actually the categories of the labelled data. This mapping of clusters into classes is illustrated in our previous work[1] and it is done by converting a cluster into a class based on the number of labelled segments within the cluster and its size (based on the number of points within the cluster) as it is shown in the equation below:

$$m_i \equiv \lceil n_i * p_{min,i} \rceil, \text{ where } , p_{min,i} \equiv max(n_i^{-\gamma}, p_{min}) \text{ and} \tag{1}$$

$$m_i \equiv \text{ minimum amount of labels } , n_i \equiv \text{cluster size}, \gamma = 0.75, \; p_{min} = 0.01 \text{ (or 1\%)}$$

Based on equation 1 smaller clusters require more labelled data in order to be assigned to a class while larger clusters require less labelled data. p_{min} acts as a threshold in the sense that is must always be 1% of labelled data available within the cluster in order to be assigned to a class disregarding its size. For example if a cluster has 100 points then $p_{min} = 0.03$ and the minimum number of labelled data needs to be $m_i = 3$. In case that the criterion described by equation 1 fails or if multiple labels of different classes are present within the cluster then the cluster is marked as undefined.

Regarding the constraints, a MUST-LINK constraint is generated between two datapoints with the same label and a CANNOT-LINK constraint is generated between two datapoints with a different label. Multilabelled datapoints are considered distinctive meaning that if for example a datapoint is labelled as *thigmotaxis* and *incursion* then a MUST-LINK constraint will be generated only with datapoints that are also labelled as *thigmotaxis* and *incursion*. In addition, a constraint is created only between relatively close datapoints, i.e. if the Euclidian distance between the two labelled datapoints is less that 0.25 (the features of the datapoints are normalized between [0 1]). The last rule has been implemented in our previous work[1] to limit the number of generated constraints since too many constraints can create computational issues.

In order to improve the classification quality we performed the 'two-stage clustering' where first we cluster the data using only the "CANNOT-LINK" constraints and then clusters that could not be mapped to a class (ambiguous clusters) are sub-divided by another clustering step, this time, however, both "CANNOT-LINK" and "MUST-LINK" constraints are used. Moreover multiple target number of clusters are tried in succession from 2 up to two times the initial number of clusters used in the first clustering. A sub-portioning is considered correct if one of the sub-clusters could be classified. The stages of this process are shown in the appendix figure 2.

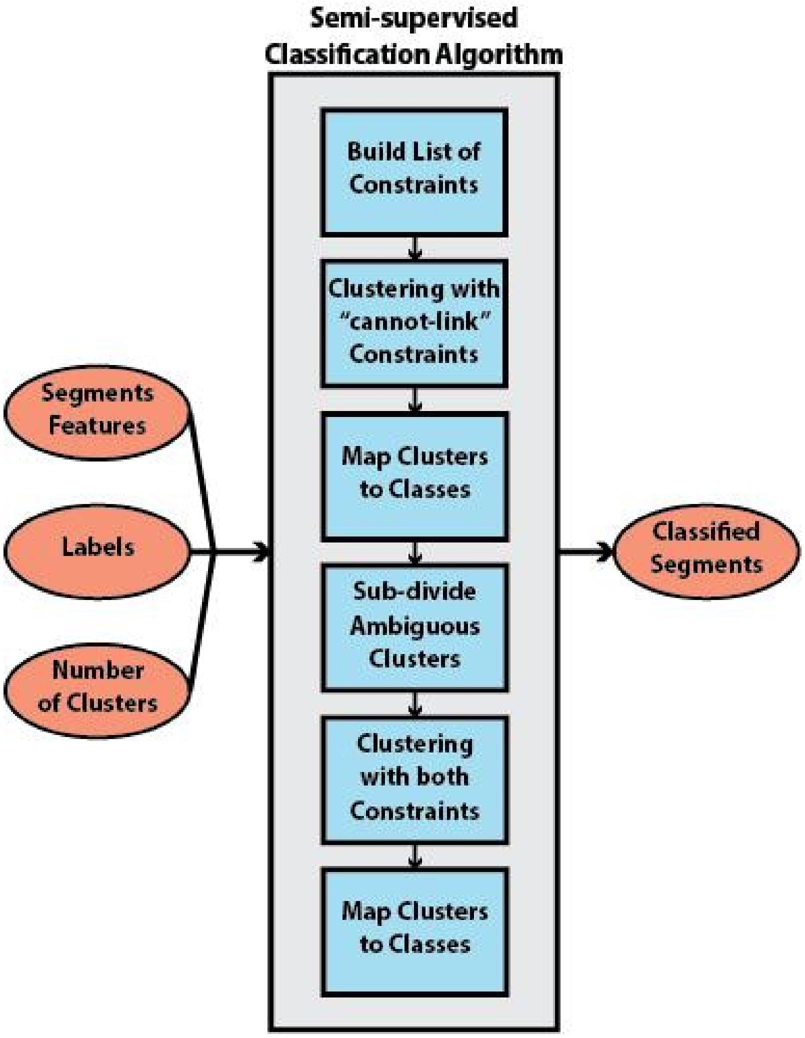

Figure S 2. Stages of the Semi-supervised Classification Algorithm. As inputs the computed features of the segments along with a partial set of labels of the segments are provided. In addition, a predefined number of target clusters needs to be provided, which specifies the number of clusters that the algorithm needs to detect. As output the algorithm provides the class in which each segment falls into. The labels are used to formed the list of constraints of which data should not be ('CANNOT-LINK') or should be ('MUST-LINK') in the same clusters. In the first-stage clustering only the 'CANNOT-LINK' constrains are used to guide the clustering procedure and then clusters that could not be mapped into classes (ambiguous clusters) are sub-divided and a second clustering stage begins this time with both CANNOT-LINK and MUST-LINK constraints.

C 10-Fold Cross Validation (for tuning and testing)

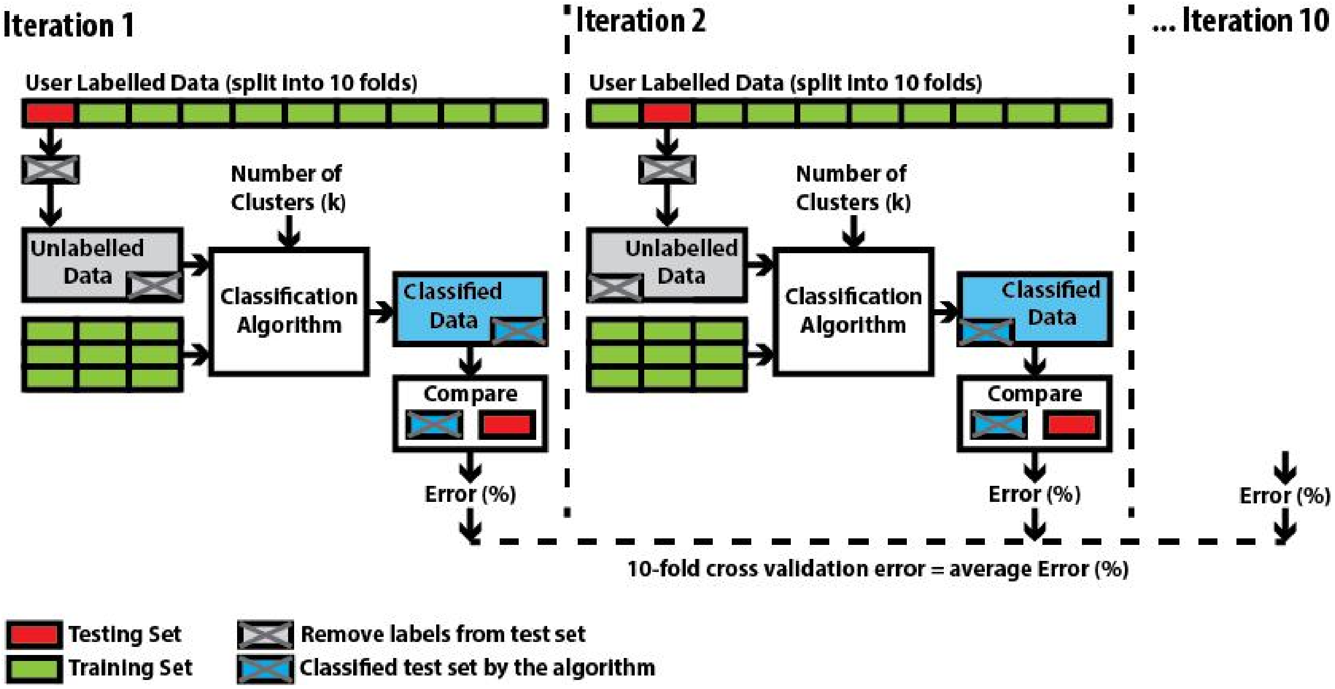

Figure S 3. 10-Fold cross validation process. The manually labelled data are split into 10 folds, 9 of which are used for training while 1 is used for testing the classification results. The classification error (expressed as a percentage) is calculated based on the differences between the unclassified and classified portions of the test set. The same process is repeated 10 times and each time the test set is changed with a different portion of the training set. The 10-fold cross validation error is the average error over all iterations. This process is used for the selection of the strong classifiers and for the estimation of the ensemble error. For the later, the same folds which were used to estimate the error of the classifiers are used to estimate the error of the ensemble. More strictly, in cross validation, the algorithm under test must be trained exclusively on the training set and do not receive any input from the test set (in our case the algorithm is trained using all the data but fewer labels). However, we use cross validation for both tuning and testing because the objective is not to create a generic classification but a specific one for the target dataset (other datasets would again require 8% to 12% labelling)[1]. The ensemble results of two segmentations (Segmentations II and IV) were manually assessed.

Table S 2. Manual estimation of ensemble error. The ensemble error was manually assessed for the segmentations II and IV. The table shows both the total error and the error among the different classes (including the average). The total manually estimated error of the ensembles is still significantly lower than the average error of the classifiers (6.3% vs 17.5% for Segmentation II and 2.8% vs 18.0% for Segmentation IV). The results of the ensembles were manually assessed for two reasons: (i) to estimate the overfitting, which is likely to be caused because the same data were used for both tuning and testing, and (ii) because our testing set was very small since a limited amount of labels were provided, the error estimation is likely to be overly optimistic.

	Segmentation II	Segmentation IV
TT error	1.4%	1.0%
IC error	8.0%	1.5%
SC error	6.6%	4.1%
FS error	6.0%	3.7%
CR error	11.5%	12.6%
SO error	11.0%	8.1%
SS error	9.6%	3.2%
ST error	2.3%	1.0%
average error	5.7%	4.4%
total error	6.3%	2.8%

D Smoothing Function

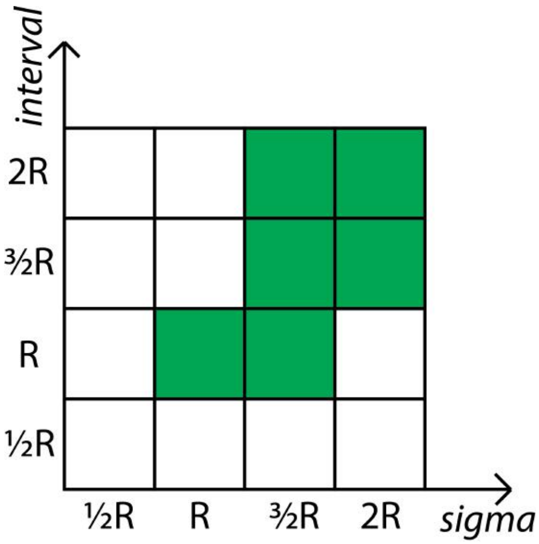

Figure S 4. Empirically defined area of tuning for the smoothing function. R refers to the arena radius (in cm); x-axis (sigma) refers to a particular value of σ (variance of the Gaussian); y-axis (interval) refers to a particular value of the length of the interval; green boxes indicate areas under which the smoothing function (refer to section 2.7 Mapping Segment Classes to the Full Swimming Paths) yields consistent results for every segmentation (excluding Segmentation I where the segments length is too large). Interval of length $2 \cdot R$ is at the limit and from this point onwards consistency cannot be sustained.

E Average Lengths of the Strategies

Table S 3. Average length of each strategy for each segmentation tuning. Each length is measured based on the arena radius R, where $R = 100$cm after the default smoothing function is applied (interval length $= R$, sigma $= R$). Tunings: Segmentation I, length $= 3 \cdot R$, overlap 70%; Segmentation II, length $= 2.5 \cdot R$, overlap 70%; Segmentation III, length $= 2.5 \cdot R$, overlap 90%; Segmentation IV, length $= 2 \cdot R$, overlap 70%. We see that the class lengths slightly differ for each segmentation tuning, however this does not affect the final conclusions. The table also shows that the lower class length that can be detected by our framework is no less than $1.9 \cdot R$.

	**Segmentation I**	**Segmentation II**	**Segmentation III**	**Segmentation IV**
**Thigmotaxis**	5.2	4.2	4.0	3.6
**Incursion**	2.5	2.3	2.3	1.9
**Scanning**	2.5	2.2	2.2	1.9
**Focused Search**	3.0	2.9	2.3	2.4
**Chaining Response**	3.8	3.2	2.7	2.3
**Scanning Surroundings**	3.0	2.5	2.3	2.1
**Target Scanning**	2.5	2.1	2.1	1.9

F Agreement Matrix

Classifiers	Classifiers																									
	1	2	3	4	5	6	7	8	9	10	11	12	13	14	15	16	17	18	19	20	21	22	23	24	...	91
1	100	61.39	36.62	46.22	39.30	34.95	44.93	49.03	37.07	45.36	48.75	44.32	44.49	34.82	47.64	45.19	44.35	41.10	41.10	40.28	38.69	35.68	38.46	45.94	...	39.01
2	61.39	100	39.87	44.28	40.06	33.67	41.38	50.01	35.47	43.82	48.02	44.63	43.07	33.10	49.25	42.49	41.29	41.26	41.26	41.93	38.74	37.26	38.27	43.07	...	40.78
3	36.62	39.87	100	50.63	54.55	53.17	41.05	37.23	49.45	38.75	41.19	35.26	38.91	46.95	42.06	40.79	41.68	40.79	40.79	41.44	48.07	47.87	43.24	42.61	...	36.66
4	46.22	44.28	50.63	100	46.25	47.10	51.14	45.10	44.65	51.04	49.85	42.63	46.79	40.52	48.84	49.48	48.56	48.77	48.77	49.98	40.38	41.40	47.61	49.14	...	44.46
5	39.30	40.06	54.55	46.25	100	54.55	45.15	43.33	51.98	39.77	41.82	37.60	38.03	48.39	41.72	40.15	36.52	40.51	40.51	41.72	46.59	48.51	43.48	39.68	...	37.48
6	34.95	33.67	53.17	47.10	54.55	100	46.23	38.06	47.88	44.68	38.89	34.48	36.26	47.70	40.21	39.81	38.73	39.61	39.61	43.10	45.54	45.86	46.72	39.36	...	37.57
7	44.93	41.38	41.05	51.14	45.15	46.23	100	43.67	40.53	47.15	43.83	40.69	43.72	40.01	45.29	45.94	46.14	46.70	46.70	48.72	40.62	42.03	47.26	44.40	...	40.97
8	49.03	50.01	37.23	45.10	43.33	38.06	43.67	100	37.31	46.71	53.86	46.17	46.87	36.05	51.12	48.57	46.12	42.14	42.14	44.88	40.99	38.67	39.14	43.34	...	44.01
9	37.07	35.47	49.45	44.65	51.98	47.88	40.53	37.31	100	48.30	38.78	32.52	32.55	45.65	40.49	39.47	40.45	42.00	42.00	42.40	44.15	49.29	42.26	40.27	...	36.47
10	45.36	43.82	38.75	51.04	39.77	44.68	47.15	46.71	48.30	100	52.48	43.91	42.74	40.83	51.29	54.69	54.55	52.60	52.60	52.86	44.93	42.87	51.15	54.85	...	48.60
11	48.75	48.02	41.19	49.85	41.82	38.89	43.83	53.86	38.78	52.48	100	53.24	50.19	39.53	53.56	46.66	46.36	44.93	44.93	49.03	38.78	35.48	42.94	47.80	...	44.48
12	44.32	44.63	35.26	42.63	37.60	34.48	40.69	46.17	32.52	43.91	53.24	100	54.00	45.74	45.45	46.31	43.91	42.40	42.40	45.14	36.21	34.57	42.39	43.54	...	42.74
13	44.49	43.07	38.91	46.79	38.03	36.26	43.72	46.87	32.55	42.74	50.19	54.00	100	39.25	50.85	48.34	47.99	44.33	44.33	46.63	39.24	37.39	39.74	44.58	...	44.21
14	34.82	33.10	46.95	40.52	48.39	47.70	40.01	36.05	45.65	40.83	39.53	45.74	39.25	100	37.91	40.45	40.05	40.16	40.16	44.44	44.85	44.83	42.67	39.30	...	39.93
15	47.64	49.25	42.06	48.84	41.72	40.21	45.29	51.12	40.49	51.29	53.56	45.45	50.85	37.91	100	52.08	53.64	49.56	49.56	51.47	44.87	43.78	48.13	52.37	...	47.13
16	45.19	42.49	40.79	49.48	40.15	39.81	45.94	48.57	39.47	54.69	46.66	46.31	48.34	40.45	52.08	100	70.70	60.17	60.17	58.03	48.61	49.53	47.45	54.12	...	47.43
17	44.35	41.29	41.68	48.56	36.52	38.73	46.14	46.12	40.45	54.55	46.36	43.91	47.99	40.05	53.64	70.70	100	58.80	58.80	58.74	52.54	50.80	49.96	58.37	...	50.39
18	41.10	41.26	40.79	48.77	40.51	39.61	46.70	42.14	42.00	52.60	44.93	42.40	44.33	40.16	49.56	60.17	58.80	100	100.0	60.64	47.34	48.82	51.50	53.96	...	50.62
19	41.10	41.26	40.79	48.77	40.51	39.61	46.70	42.14	42.00	52.60	44.93	42.40	44.33	40.16	49.56	60.17	58.80	100.0	100	60.64	47.34	48.82	51.50	53.96	...	50.62
20	40.28	41.93	41.44	49.98	41.72	43.10	48.72	44.88	42.40	52.86	49.03	45.14	46.63	44.44	51.47	58.03	58.74	60.64	60.64	100	49.62	49.92	55.04	55.79	...	51.98
21	38.69	38.74	48.07	40.38	46.59	45.54	40.62	40.99	44.15	44.93	38.78	36.21	39.24	44.85	44.87	48.61	52.54	47.34	47.34	49.62	100	64.70	51.90	50.11	...	42.72
22	35.68	37.26	47.87	41.40	48.51	45.86	42.03	38.67	49.29	42.87	35.48	34.57	37.39	44.83	43.78	49.53	50.80	48.82	48.82	49.92	64.70	100	48.68	49.35	...	41.14
23	38.46	38.27	43.24	47.61	43.48	46.72	47.26	39.14	42.26	51.15	42.94	42.39	39.74	42.67	48.13	47.45	49.96	51.50	51.50	55.04	51.90	48.68	100	60.08	...	47.97
24	45.94	43.07	42.61	49.14	39.68	39.36	44.40	43.34	40.27	54.85	47.80	43.54	44.58	39.30	52.37	54.12	58.37	53.96	53.96	55.79	50.11	49.35	60.08	100	...	52.84
...	...	...	...	...	...	...	...	...	...	...	...	...	...	...	...	...	...	...	...	...	...	...	...	...	100	...
91	39.01	40.78	36.66	44.46	37.48	37.57	40.97	44.01	36.47	48.60	44.48	42.74	44.21	39.93	47.13	47.43	50.39	50.62	50.62	51.98	42.72	41.14	47.97	52.84	...	100

Figure S 5. Agreement matrix for the classifiers of Segmentation III. The classifier of each column is being compared with the classifier of each row. The comparison is based on the percentage of segments which both classifiers agree belong to the same class. The diagonal values of the matrix indicate 100% agreement since each classifier is compared with itself.

G Results of each Segmentation without the Smoothing Function

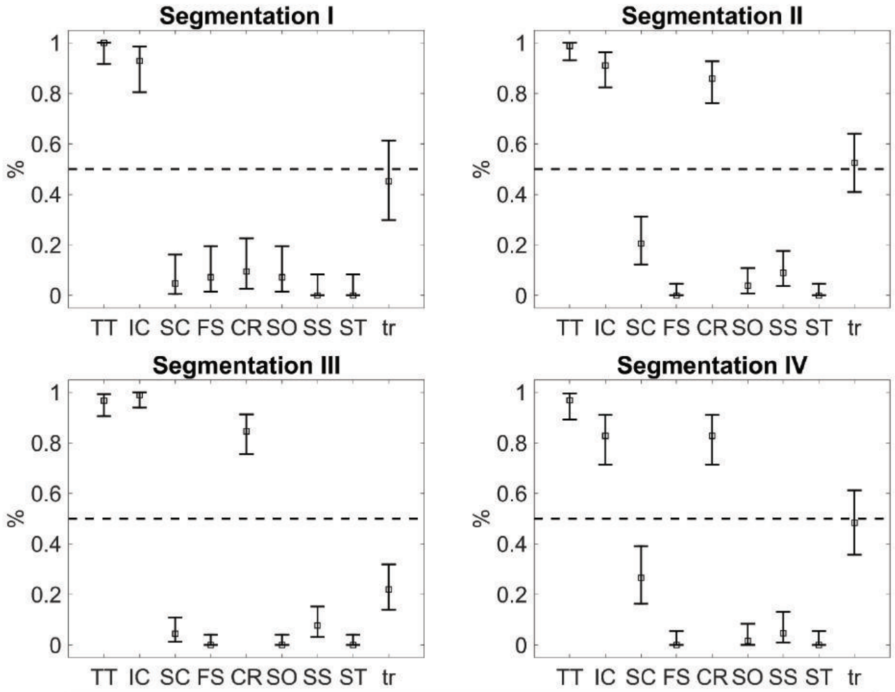

Ensemble Result<br>Friedman test p-values and Q per strategy and transitions (k=2)									
**p-val**	**TT**	**IC**	**SC**	**FS**	**CR**	**SO**	**SS**	**ST**	**tr**
**I**	**0.008**	**0.007**	0.469	0.196	0.159	0.724	0.240	0.375	0.061
**II**	**0.004**	**0.007**	0.165	0.355	**0.006**	0.756	0.201	0.284	0.066
**III**	**0.008**	**0.007**	0.335	0.946	**0.012**	0.844	0.220	0.584	0.161
**IV**	**0.012**	**0.014**	0.178	0.900	**0.006**	0.895	0.237	0.301	**0.035**
Q	***TT***	***IC***	***SC***	***FS***	***CR***	***SO***	***SS***	***ST***	***tr***
I	***7.100***	***7.263***	*0.525*	*1.670*	*1.982*	*0.125*	*1.380*	*0.787*	*3.505*
II	***8.152***	***7.248***	*1.932*	*0.856*	***7.443***	*0.097*	*1.632*	*1.150*	*3.388*
III	***6.973***	***7.417***	*0.929*	*0.005*	***6.382***	*0.039*	*1.504*	*0.300*	*1.964*
IV	***6.367***	***5.984***	*1.815*	*0.016*	***7.715***	*0.018*	*1.401*	*1.071*	*4.424*

Figure S 6. Conclusive pre-smoothing results from the classification of each segmentation Considering segments as continuous parts of the trajectories ignoring the overlapping provides consistent results when differences between the implemented strategies of the groups are being investigated but creates an overestimation on the number of transitions between strategies. Each plot shows the 95% binomial confidence intervals for the classifiers of each segmentation regarding their agreement if there is significant difference between the two animal groups (i.e Friedman test p-value < 0.05) on each strategy and strategy transitions or not. Squares indicate the mean of the classifiers that shows that there is a significant difference in this particular case; errorbars are the 95% confidence intervals; the dashed line indicates the threshold of interest (0.5 or 50%). The table below the plots shows the Friedman test p-values (upper table) and the equivalent Friedman's chi-square statistic (lower table) for the classification result of the ensemble; in all cases $k = 2$, control and stress columns. Segmentation configurations are arranged in columns and strategies in rows; each element has the relevant p-value and chi-square statistic and bold cells indicate significant difference, i.e. p-value < 0.05. In order to be confident that there is indeed a significant difference between the two animal groups on each strategy and the strategy transitions the confidence intervals should be clearly above 0.5 (or 50%). Compared to the results in the main manuscript, we see that the smoothing function which maps the segments to the full swimming paths is actually beneficial for revealing the animal transitions between strategies. Other than that, the results lead to the same conclusions.

Table S 4. Classification statistics for the four segmentation configurations prior to smoothing. In comparison with the results of the main manuscript we see that the percentage of unclassified segments among the classifiers is higher and the agreement between them lower. However, the ensemble (or ensembles in case of the agreement) again nearly nullifies the unclassified segments and significantly boosts the agreement percentage.

	Segmentation I	Segmentation II	Segmentation III	Segmentation IV
	Classifiers			
Unclassified (%) Segments	24.8	24.3	30.0	29.0
Agreement (%)	53.2	55.5	48.8	52.1
	Ensemble(s)			
Unclassified (%) Segments	1.2	0.7	0.8	1.1
Agreement (%)	84.7	83.3	79.8	80.0

H Ensemble Results of each Segmentation

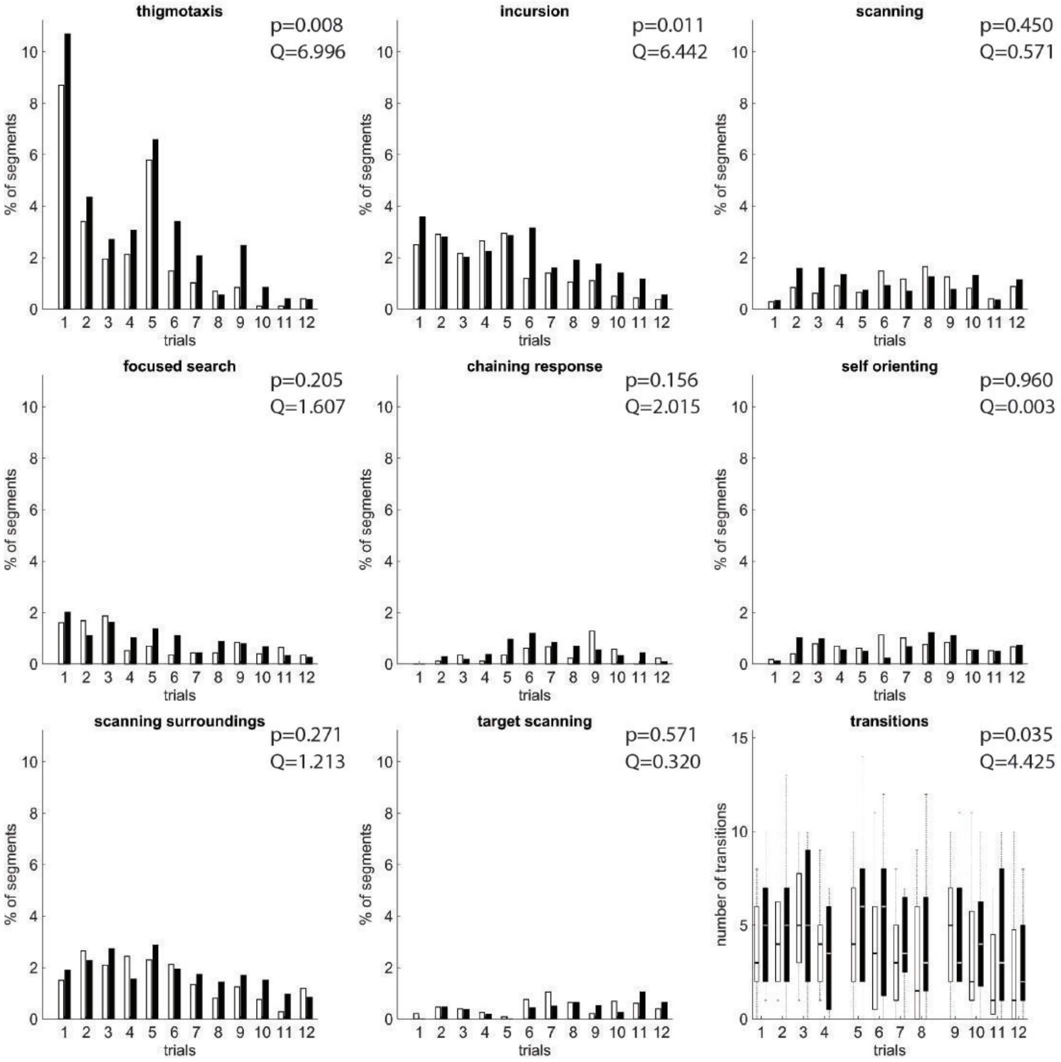

Figure S 7. Percentage of segments falling under each strategy for the stressed (black) and control (white) animal groups over each trial for the Segmentation I. All the animals were tested for a set of 12 trials divided in 3 sessions (days). Each segment is considered to be of a length equal to the arena radius (100cm). For the transitions: bars represent the first and third quartiles of the data; the black (control group) or white (stressed group) horizontal lines denotes the median, crosses are the outliers and whiskers indicate the minimum and the maximum values. The Friedman test (shown on the top right) was used to compare both animal groups for the complete set of trials. According to the plots, stressed animals produce longer paths since the average number of strategy implementations is higher than in the control group. Thigmotaxis (Friedman test p-value $= 0.008$, $Q = 6.996$, $k = 2$) and Incursion (Friedman test p-value $= 0.011$, $Q = 6.442$, $k = 2$) strategies show a clear difference in favor of the stressed group along with the strategy transitions (Friedman test p-value $= 0.035$, $Q = 4.425$, $k = 2$). This Segmentation configuration fails to reveal significant differences on the Chaining Response because of the segment length which causes some rarer strategies to disappear.

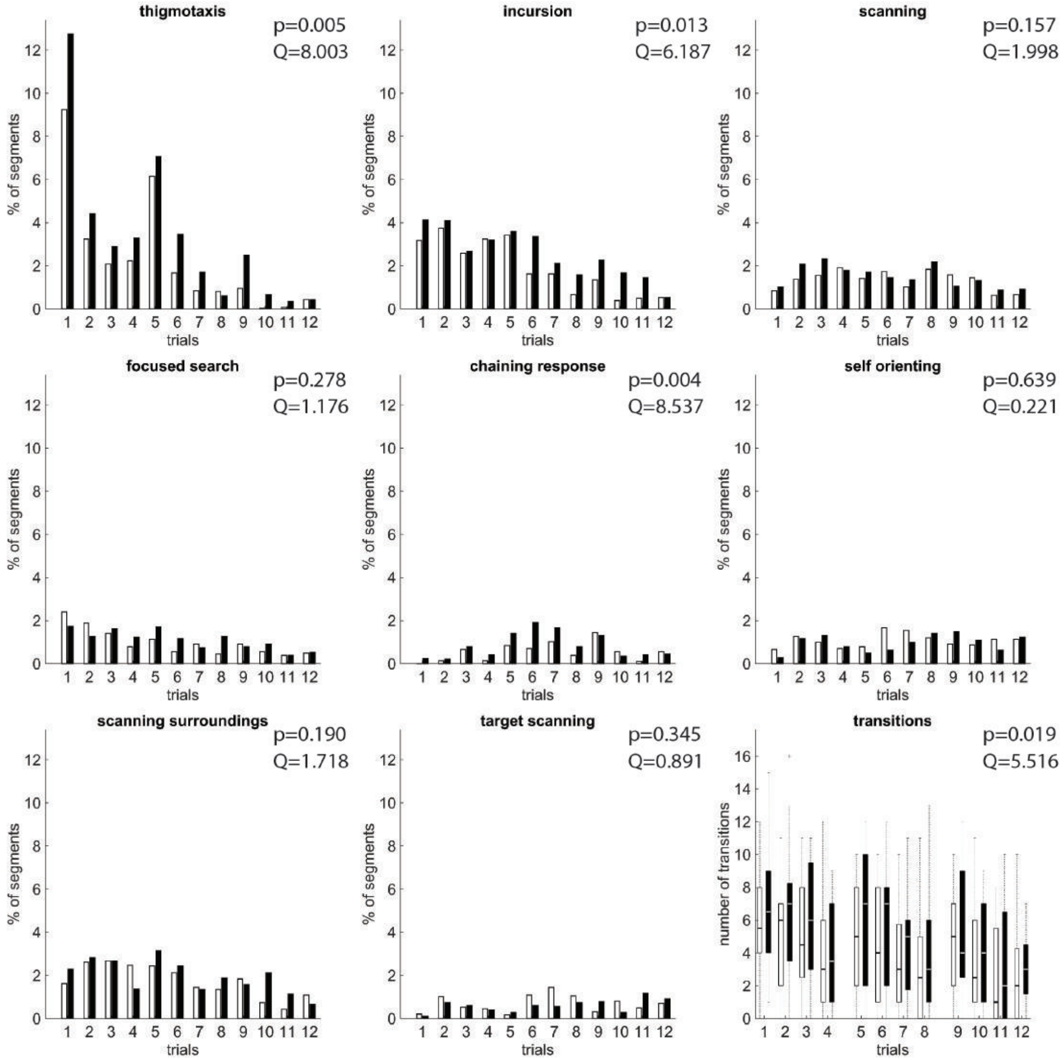

Figure S 8. Percentage of segments falling under each strategy for the stressed (black) and control (white) animal groups over each trial for the Segmentation II. All the animals were tested for a set of 12 trials divided in 3 sessions (days). Each segment is considered to be of a length equal to the arena radius (100cm). For the transitions: bars represent the first and third quartiles of the data; the black (control group) or white (stressed group) horizontal lines denotes the median, crosses are the outliers and whiskers indicate the minimum and the maximum values. The Friedman test was used to compare both animal groups for the complete set of trials. According to the plots, stressed animals produce longer paths since the average number of strategy implementations is higher than in the control group. Thigmotaxis (Friedman test p-value $= 0.005$, $Q = 8.003$, $k = 2$) and Incursion (Friedman test p-value $= 0.013$, $Q = 6.187$, $k = 2$) strategies show a clear difference in favor of the stressed group along with Chaining Response (Friedman test p-value $= 0.004$, $Q = 8.537$, $k = 2$). The number of transitions between strategies shows that the stressed animals change their behaviour more often within single trials (Friedman test p-value $= 0.019$, $Q = 5.516$, $k = 2$).

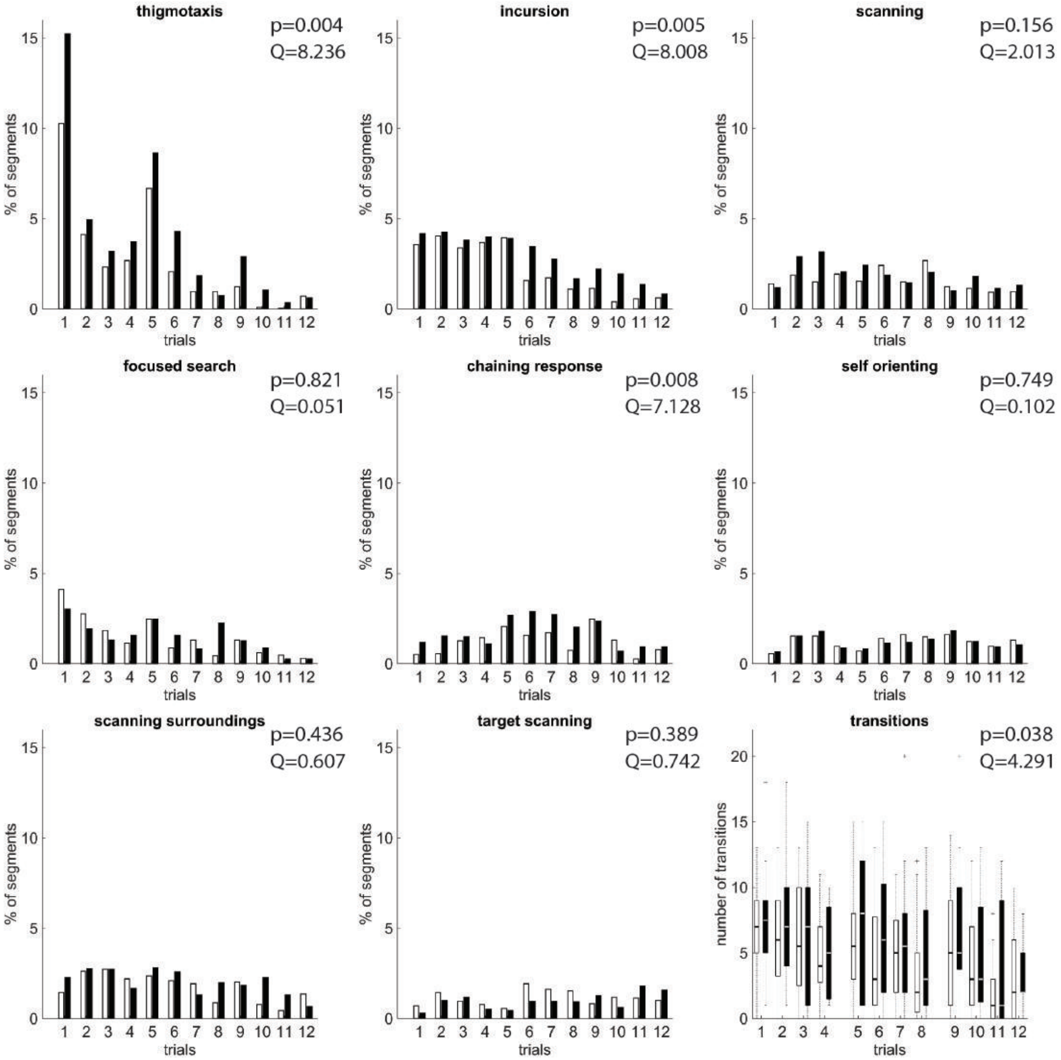

Figure S 9. Percentage of segments falling under each strategy for the stressed (black) and control (white) animal groups over each trial for the Segmentation IV. All the animals were tested for a set of 12 trials divided in 3 sessions (days). Each segment is considered to be of a length equal to the arena radius (100cm). For the transitions: bars represent the first and third quartiles of the data; the black (control group) or white (stressed group) horizontal lines denotes the median, crosses are the outliers and whiskers indicate the minimum and the maximum values. The Friedman test p-value and chi-square statistic (shown on the top right) was used to compare both animal groups for the complete set of trials. According to the plots, stressed animals produce longer paths since the average number of strategy implementations is higher than in the control group. Thigmotaxis (Friedman test p-value $= 0.004$, $Q = 8.236$, $k = 2$) and Incursion (Friedman test p-value $= 0.005$, $Q = 8.008$, $k = 2$) strategies show a clear difference in favor of the stressed group along with Chaining Response (Friedman test p-value $= 0.008$, $Q = 7.128$, $k = 2$). The number of transitions between strategies shows that the stressed animals change their behaviour more often within single trials (Friedman test p-value $= 0.038$, $Q = 4.291$, $k = 2$).

References

1. Gehring, T. V., Luksys, G., Sandi, C. & Vasilaki, E. Detailed classification of swimming paths in the morris water maze: multiple strategies within one trial. *Sci. reports* **5** (2015).
2. Gärtner, B. & Schönherr, S. Smallest enclosing ellipses–fast and exact. *CiteSeerx* (1997).
3. Bilenko, M., Basu, S. & Mooney, R. J. Integrating constraints and metric learning in semi-supervised clustering. In *Proceedings of the twenty-first international conference on Machine learning*, 11 (ACM, 2004).
4. Jain, A. K. Data clustering: 50 years beyond k-means. *Pattern recognition letters* **31**, 651–666 (2010).
